# Structure and Dynamics of the Membrane-Bound Cytochrome P450 2C9

**DOI:** 10.1371/journal.pcbi.1002152

**Published:** 2011-08-11

**Authors:** Vlad Cojocaru, Kia Balali-Mood, Mark S. P. Sansom, Rebecca C. Wade

**Affiliations:** 1 Molecular and Cellular Modeling Group, Heidelberg Institute for Theoretical Studies, Heidelberg, Germany; 2 Department of Cell and Developmental Biology, Max Planck Institute for Molecular Biomedicine, Münster, Germany; 3 Structural Bioinformatics and Computational Biochemistry Unit, Department of Biochemistry, University of Oxford, Oxford, United Kingdom; University of California San Diego, United States of America

## Abstract

The microsomal, membrane-bound, human cytochrome P450 (CYP) 2C9 is a liver-specific monooxygenase essential for drug metabolism. CYPs require electron transfer from the membrane-bound CYP reductase (CPR) for catalysis. The structural details and functional relevance of the CYP-membrane interaction are not understood. From multiple coarse grained molecular simulations started with arbitrary configurations of protein-membrane complexes, we found two predominant orientations of CYP2C9 in the membrane, both consistent with experiments and conserved in atomic-resolution simulations. The dynamics of membrane-bound and soluble CYP2C9 revealed correlations between opening and closing of different tunnels from the enzyme's buried active site. The membrane facilitated the opening of a tunnel leading into it by stabilizing the open state of an internal aromatic gate. Other tunnels opened selectively in the simulations of product-bound CYP2C9. We propose that the membrane promotes binding of liposoluble substrates by stabilizing protein conformations with an open access tunnel and provide evidence for selective substrate access and product release routes in mammalian CYPs. The models derived here are suitable for extension to incorporate other CYPs for oligomerization studies or the CYP reductase for studies of the electron transfer mechanism, whereas the modeling procedure is generally applicable to study proteins anchored in the bilayer by a single transmembrane helix.

## Author Summary

We describe the first atomic-detail models and simulations of a full-length, membrane-bound mammalian cytochrome P450. To date, all the structural studies of microsomal, drug-metabolizing cytochrome P450s have been performed using engineered, solubilized forms of the enzymes and it is not yet understood how the membrane influences their structure, dynamics, and ability to bind substrates. We focused on CYP2C9, the second most abundant cytochrome P450 in the human liver which oxidizes 20% of all marketed drugs. Here, we have derived models of CYP2C9-membrane complexes with a modeling procedure based on molecular dynamics simulations started with arbitrary configurations of the protein in the membrane and performed using both coarse grained and atomic-detail molecular representations. This procedure is expected to be generally applicable to proteins that are anchored in the membrane with a single transmembrane helix. The models and simulations provide evidence for selective substrate access and product release routes in membrane-bound CYPs. This observation may contribute to new strategies to manipulate the activity of CYPs and other enzymes with buried active sites. We anticipate that this study will bring about a paradigm shift towards studying microsomal CYPs as dynamic structures in their natural, lipid environment rather than in artificially solubilized forms.

## Introduction

The liver-specific, mammalian cytochromes P450 (CYPs) are monooxygenases essential for drug metabolism [1]. CYPs accept two electrons from the CYP reductase (CPR) to oxidize a wide range of water-soluble and liposoluble xenobiotics. CYPs and the CPR are anchored in the membrane of the endoplasmic reticulum (ER) by a single transmembrane anchor with the catalytic domain facing the cytosol [2]. The phospholipid composition has been shown to modulate different steps of catalysis [3], ligand binding [4] and the binding of CYPs to CPR [5]. The interaction of CYPs with the membrane has been suggested to permit the direct binding of liposoluble substrates to the enzyme's buried active site [6], [7]. Therefore, it is essential to understand this interaction at atomic resolution.

CYPs are anchored in the membrane by a single N-terminal transmembrane α-helix. In addition, CYPs insert a hydrophobic region of the catalytic domain in the lipid bilayer. This has been shown by experiments that probed the recognition of reconstituted microsomes by site-directed antibodies against peptides of the human CYP2B1 and the rabbit CYP2B4 [2], [8]. Engineered CYPs with the transmembrane domain removed retain membrane-binding properties [2], [9]. The height of the globular domain above the lipid bilayer has been estimated to be 35±9 Å [10]. The heme tilt angle with respect to the membrane (the dihedral angle between the heme plane and the membrane plane) has been shown to vary in different CYP isoforms from 38 to 78° [11]. The positions of different residues of CYP2C2 relative to the membrane have been inferred from tryptophan fluorescence quenching experiments [12]. These experiments provide valuable information for building models of the membrane-bound CYPs. However, not all the experiments are consistent with each other and there are multiple, different orientations of the protein in the membrane that are consistent with different experiments. Moreover, if different CYPs actually adopt different orientations in the membrane, the relevance of the experiments might be restricted to the investigated isoform.

Based on the first crystal structure of a mammalian CYP, the rabbit CYP2C5, a model with a shallow insertion of the catalytic domain in the membrane was proposed [13]. From the crystal structure of human CYP2C8 as a dimer, a model for CYP oligomerization in the membrane was suggested [14]. Follow-up experiments confirmed the dimeric state of CYP2C8 [15]. From these two models, the approximate orientation of CYPs in the membrane was proposed. The estimated values of the heme tilt angle were in the upper range of the experimentally derived values [11]. However, these models have not been investigated further by other approaches such a computer simulation. Furthermore, such models are limited by the uncertainties in the atomic resolution structures of CYPs which were resolved using engineered enzymes with the N-terminal transmembrane helix removed. In some cases, protein solubility was further increased by mutation in the region of the FG loop [7]. Computational tools such as the OPM (Orientations of Proteins in Membranes) database fail to predict an orientation of CYPs in the membrane that is consistent with experiments [16]. Therefore, an atomic-resolution model of the CYP-membrane interaction remains elusive.

Most of the crystal structures of CYPs reveal a closed conformation of the enzymes. Several structures of the rabbit CYP2B4 show protein conformations with wide open clefts that were proposed to be relevant in the context of lipid bilayers [17], [18]. In addition, the human CYP3A4 has been shown to adopt open conformations depending on the size of the bound substrate [19]. To what extent the membrane influences the ability of CYPs to adopt open conformations is not understood.

The CYP active site is buried deep in the protein. Multiple tunnels from the active site were found in CYP crystal structures and have been classified [20]. From simulations of ligand egress, it was proposed that mammalian CYPs may use more than one tunnel for ligand passage. It was suggested that liposoluble substrates bind through tunnels leading from the lipid bilayer while soluble products are released through solvent-accessible routes [6], [21], [22]. How the lipid bilayer influences the opening and closing of different tunnels remains unclear.

Here, we report the structure and dynamics of the full-length, membrane-bound CYP2C9, the second most abundant CYP in the human liver which metabolizes about 20% of all xenobiotics [23]. We found two orientations of CYP2C9 in the membrane, both consistent with experiments. While the enzyme remained mostly in closed or nearly closed conformations, we found both membrane and ligand dependent correlations between opening and closing of different tunnels from the buried catalytic center. We propose that the membrane promotes binding of liposoluble substrates by stabilizing protein conformations with an open access tunnel and provide evidence for selective substrate access and product release routes in mammalian CYPs. We describe a procedure to model and simulate the membrane-bound CYPs that is not biased towards a certain orientation of the protein in the membrane and that may be extended to incorporate the CPR as well.

## Results

### Simulations reveal two predominant orientations of CYP2C9 in the membrane

**Figure 1:**
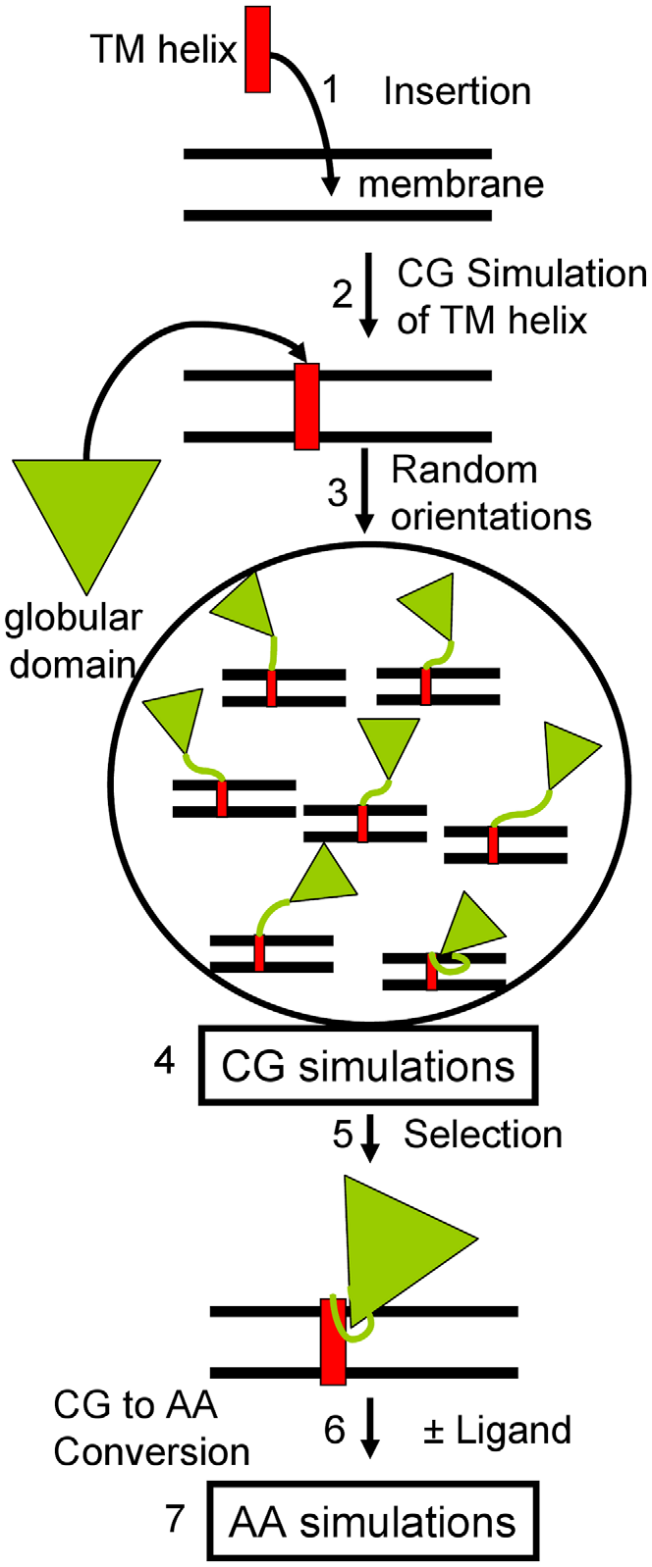
Procedure to model and simulate the membrane-bound CYP2C9. (1) The transmembrane N-terminal helix (red rectangle) was inserted in a lipid bilayer; (2) The system was simulated for 2 µs with a coarse grained force field for proteins and lipids; (3) The globular domain of CYP2C9 (green triangle) was added in different orientations by randomly changing dihedral angles in the linker peptide; (4) 1 µs of coarse grained simulation was performed for each model; (5) From the analysis of the CYP2C9 orientation in the membrane during the coarse grained simulations, protein-membrane configurations were selected, converted to atomic resolution (6) and used in follow-up atomic-resolution simulations (7).

We applied a procedure based on multiple coarse grained simulations (Fig. 1, Steps 1–5) followed by atomic-resolution (Fig. 1, Steps 6–7) simulations to study the structure and dynamics of CYP2C9 in a 1-palmitoyl-2-oleoylphosphatidylcholine (POPC) bilayer. POPC was chosen because it is one of the major components of the endoplasmic reticulum membrane (>50% phosphatidylcholine [24]). An initial bias towards a specific orientation of CYP2C9 in the membrane was prevented by starting the coarse grained simulations with randomized orientations of the globular domain above or slightly inserted in the membrane. We simulated models derived from two conformations of CYP2C9 (Table 1), both based on the flurbiprofen-bound crystal structure (pdb 1R9O) [25]. 1R9OH1 and 1R9OH2 are full-length models while 1R9O1 and 1R9O2 represent soluble forms with the transmembrane helix removed. In the FG loop, which is thought to dip into the membrane, 1R9O1 and 1R9OH1 have two short helices (F' and G') while 1R9O2 and 1R9OH2 have an extended structure. The nomenclature for the CYP secondary structure is from [26].

**Table 1:** Summary of atomic-resolution simulations of CYP2C9 models.

Model	Properties^2^	Time (ns)	Ensemble (γ dyn/cm)	Area per lipid (Å^2^)
1R9O1	SOL^2^, ^a^F'G' helices^2^ ^b^, APO^2^ ^c^	28.28	NPT	-
1R9O1+FLO^1^	SOL, F'G' helices, PROD^2^ ^c^	27.84	NPT	-
1R9O2	SOL, FG loop^2^ ^b^, APO	27.78	NPT	-
1R9O2+FLO	SOL, FG loop, PROD	27.4	NPT	-
1R9O2+FLU^1^	SOL, FG loop, SUBS^2^ ^c^	25.98	NPT	-
1R9OH1+POPC	MEM^2^ ^a^, F'G' helices, APO	11.25	NPγT (γ = 50)	64.13±0.43
		18.75	NPγT (γ = 60)	67.08±0.96
1R9OH1+FLO+POPC	MEM, F'G' helices, PROD	11.25	NPγT (γ = 50)	64.43±0.58
		18.75	NPγT (γ = 60)	69.23±1.16
1R9OH2+POPC	MEM, FG loop, APO	11.25	NPγT (γ = 50)	62.02±1.03
		18.75	NPγT (γ = 60)	64.09±0.73
1R9OH2+FLO+POPC	MEM, FG loop, PROD	11.25	NPγT (γ = 50)	62.76±0.59
		18.75	NPγT (γ = 60)	65.67±0.66

1. FLO = 4′-hydroxy-flurbiprofen, FLU  =  Flurbiprofen.

2. The following properties differ among the simulated CYP2C9 models.

a. Solubility (water soluble ‘SOL’ and membrane-bound ‘MEM’ forms).

b. Structure of the FG loop (with F' and G' helices or extended loop).

c. Ligand-bound state (apo ‘APO’, product-bound ‘PROD’ and substrate-bound ‘SUBS’ forms).

In the coarse grained simulations, the equilibration of the systems was reached rapidly (∼10–20 ns). The root mean square deviation of the globular domain of CYP2C9 relative to the initial structure reached an equilibrium value that varied between 2.8 and 3.5 Å depending on the simulated system (Fig. S1).

**Figure 2:**
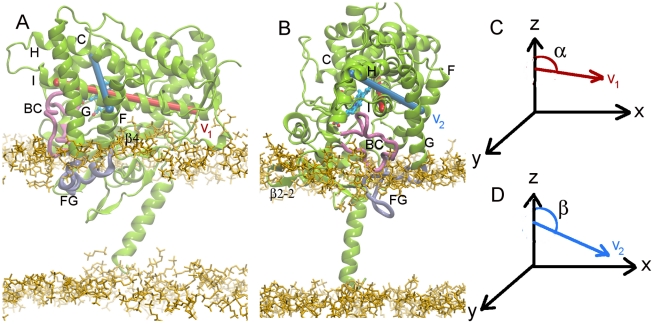
Models of membrane-bound CYP2C9. (A) The 1R9OH1 model with the F' and G' helices in the FG loop. (B) The 1R9OH2 model with the FG loop unstructured. In order to define the orientation of the protein in the membrane, the complex was positioned with the membrane in the xy plane and vectors **v_1_** and **v_2_** were defined as follows: **v_1_** (red): along the I helix, connecting the centers of the first and last helical turns in helix I defined by the midpoints of the C_α_ atoms of residues 285–289 and 312–316 respectively, and **v_2_** (blue), orthogonal to **v_1_**, connecting one helical turn in helix C and one in helix F, i.e. the midpoints of the C_α_ atoms of residues 127–131 and 197–201, respectively. The orientation of CYP2C9 in the membrane was defined by the angles α (C) and β (D) between **v_1_** and **v_2_** and the z axis. The protein is shown in green cartoon representation with the FG and BC loops highlighted in cyan and mauve respectively. The heme is shown in a stick representation colored by atom type. The secondary structure is labeled as follows: helices with letters, strands with numbers, and loops with the labels of the 2 adjacent helices or sheets. The lipid head groups are shown in yellow. These protein and lipid representations are used throughout this manuscript.

To specify the orientation of the protein in the membrane, we defined three parameters: the distance between the centers of mass of the protein and the membrane (d), and the two angles between the z axis and the vectors **v_1_** (α) and **v_2_** (β), see Fig. 2 for definition. Angles α and β are suitable descriptors of the orientation of the protein in the membrane because their fluctuations due to internal protein dynamics are negligible (Fig. S2).

**Figure 3:**
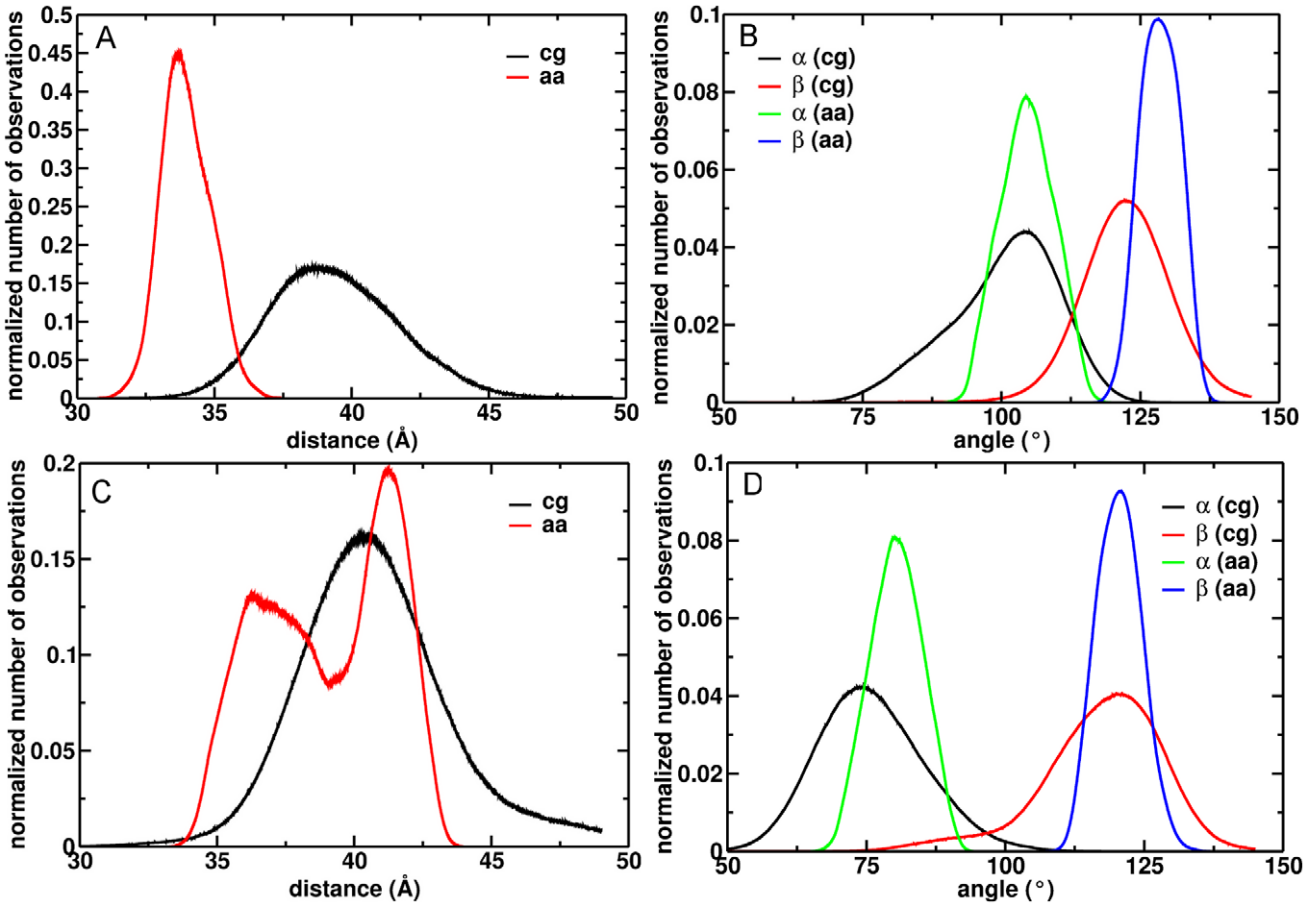
Predominant orientations of CYP2C9 in the membrane. The projection of the distance between the protein and membrane centers of mass on the z axis is shown for the 1R9OH1 (A) and 1R9OH2 (C) models during the coarse grained (black) and atomic-resolution simulations (red). The angles α and β that define the protein orientation in the membrane (Fig. 2) are shown for the 1R9OH1 (B) and 1R9OH2 (D) models during the coarse grained (black and red) and atomic-resolution (green and blue) simulations.

From the coarse grained simulations of the membrane-bound 1R9OH1 and 1R9OH2 models, we found two predominant orientations of CYP2C9 in the bilayer depending on the conformation of the FG loop. The orientations are described by the following parameters: (i) d = 39.5±2.5 Å, α = 100±9°, β = 123±8°, and (ii) d = 40.9±3.0 Å, α = 76±9°, β = 117±11° (Fig. 3). 1R9OH1 inserted slightly further into the membrane and was oriented with the end of helix I closer to the lipid with **v_1_** pointing towards the membrane (α>90°) (Fig. 2A), while 1R9OH2 oriented with the beginning of helix I closer to the lipid and **v_1_** pointing away from the membrane (α<90°) (Fig. 2B). For each membrane-bound model, one snapshot with the orientation parameters (d, α, β) within 1% of the peak values of the corresponding histograms (Fig. 3) was selected for atomic-resolution simulations.

Atomic-resolution simulations were performed for product-bound and apo forms of the membrane-bound 1R9OH1 and 1R9OH2 and of the soluble 1R9O1 and 1R9O2 models (Table 1). In addition, a substrate-bound 1R9O2 model was simulated. The systems with soluble models of CYP2C9 were equilibrated for 2.5 ns, whereas the systems with membrane-bound models were equilibrated for 9 ns (Fig. S3). The orientations of CYP2C9 in the membrane were generally conserved, although small deviations from the starting configurations were observed (Fig. 3). The consistency with experiments was good (Table 2).

**Table 2:** Consistency of the membrane-bound models of CYP2C9 with experimental data.

Experimental observable^1^	1R9OH1^2^ *		1R9OH2^2^ *		Exp. result	Exp. CYP	Exp.type
1–30	M^3^	+	M	+	IA^5^	2B1	site-directed antibody 7 ^a^
17–28	M-HG^3^-C3,b	0	M-HG-C	+	A^5^	2B4	„ 7 ^b^
23–37	C-HG-M^b^	0	C-HG-M	+	A	2B1	„ 7 ^a^
39–47	M-HG	+	M-HG-C	0	IA	2B1	„ 7 ^a^
60–71^4^	M	-	HG-M	-	A	2B1	„ 7 ^a^
92–97^4^	C	-	C	-	IA	2B1	„ 7 ^a^
107–115	C	+	C	+	A	2B1	-„ 7 ^a^
121–130	C	+	C	+	A	2B1	„ 7 ^a^
185–192	C	+	C	+	A	2B1	„ 7 ^a^
210–222	HG-M	+	HG-M	+	IA	2B1	„ 7 ^a^
224–231	M-HG-C	0	HG-C	+	A	2B1	„ 7 ^a^
314–322	C	+	C	+	A	2B1	„ 7 ^a^
397–407	C	+	C	+	A	2B1	„ 7 ^a^
36, 69^6^	M	+	M	+	M	2C2	TRP fluorescence quenching 7 ^c^
380^6^	HG	-	M	0	M	2C2	“ 7 ^c^
80, 120, 189, 239, 347	C	+	C	+	C (HG)	2C2	“ 7 ^c^
225^6^	HG-M	0	HG-C	+	C (HG)	2C2	“ 7 ^c^
height above membrane	33.6±0.7 Å	+	41.0±1.0 Å	+	35±9 Å	2B4	atomic force microscopy 7 ^d^

1. For experiments based on site-directed antibodies and Trp fluorescence quenching, the experimental observable is shown as the residue numbers mapped on CYP2C9 based on sequence alignment with the CYP used in the experiment.

2. The structures corresponding to the maximum peaks of the orientation histograms (Fig. 3) were compared with experiments.

3. The location of protein residues with respect to the membrane is classified as: (i) M: lipid tail region of the membrane, (ii) HG: the head group region, (iii) C: the cytosol. For each peptide, the location in the models was assessed starting from the N-terminal residue (e.g. a peptide with its location identified as ‘M-HG-C’ has its N-terminal residues in the membrane and the C-terminal residues in the cytosol).

4. Peptides for which the location in the models does not agree with the location inferred from the experiments.

5. The peptides were either accessible (A) or inaccessible (IA) when site-directed antibodies were tested on microsomes.

6. From the experiments based on TRP fluorescence quenching, it was inferred that residue 380 is the deepest buried in the membrane, residues 36 and 69 are in the membrane but not as deep as 380, while residues 80 and 225 are in the region of the head groups or do not contact the membrane (these 2 options are indistinguishable in the experiments). In the simulations, residue 380 is either in the head group region or lies shallow in the membrane, while residue 225 is either positioned shallow in the membrane or in the head group region.

b. The peptide is not in contact with the membrane but is buried in the protein globular domain.

*The agreement with experiment was assessed as follows: (i) ‘+’  =  good, (ii) ‘0’  =  possible, (iii) ‘-’  =  disagreement.

7. (a) [2]; (b) [8]; (c) [11]; (d) [10].

The percentage of the catalytic domain in contact with the lipid bilayer was between 24–26% (extended FG loop) and 32–34% (helical FG loop) (Table S1). The helix A, the β1 sheet, and the FG loop were in contact with the lipid bilayer in all cases. The extended FG loop inserted deeper in the membrane than the F' and G' helices. The residues at the N-terminus of the G helix were located in the proximity of the lipid head groups except for in the simulations of the ligand-free 1R9OH2 model in which they inserted in the membrane. For this model, a decrease of d was observed during the atomic-resolution simulations (Fig. 3C, red). The BC loop was accessible to the solvent except for residues 101–104 which were close to the lipid head groups, independent of the FG loop conformation. In the simulations of the structures derived from 1R9OH1, the C-terminal region was in contact with the lipid head groups; this observation accounts for the higher percentage of residues contacting the membrane in this model. As expected, and not just because of the presence of the transmembrane helix, we found that hydrophobic aminoacids penetrate deeper in the lipid bilayer whereas charged protein residues interact preferentially with the lipid head group (Fig. S4). All residues interacting with the lipids are shown in Fig. S5.

### The membrane exerts a limited influence on the flexibility of CYP2C9

**Figure 4:**
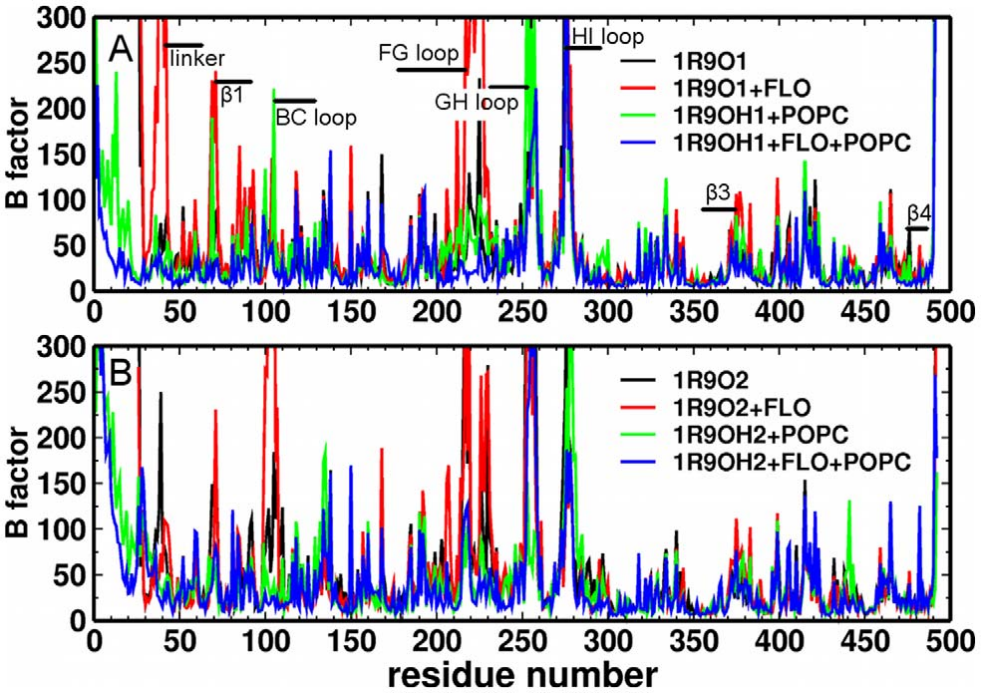
CYP2C9 flexibility. The average B factors (mean squared atomic positional fluctuations multiplied by 8π^2^/3) in Å^2^ during the atomic-resolution simulations of models are plotted for each residue: soluble 1R9O1 (A, black and red), membrane-bound 1R9OH1 (A, green and blue), soluble 1R9O2 (B, black and red), and membrane-bound1R9OH2 (B, green and blue) (in each case, with and without product (FLO) bound).

Comparing the longer atomic-resolution simulations (with γ = 60 dyn/cm) of the membrane-bound models with the simulations of the soluble models, we found that the lipid bilayer exerted a limited influence on protein flexibility (Fig. 4). The most flexible regions were the BC, FG, GH, and HI loops. The fluctuations in the BC and FG loops, and the β1 were reduced by the presence of the lipid bilayer, while the flexibility of the GH and HI loops was not affected. This is consistent with the positions of these loops relative to the membrane. The FG loop is inserted in the membrane and some residues in the BC loop are in contact with lipids, while the GH and HI loops are not in the proximity of the membrane.

The CYP2C9 models remained in closed or nearly-closed conformations in all the simulations. This is reflected in the low root mean square deviation values of the simulation snapshots relative to a reference structure chosen for each system after the initial energy minimization (Fig. S6A,B). Large opening motions such as those identified from CYP2B4 crystal structures [17], [27] were not observed. However, we observed opening-closing motions of discrete tunnels from the enzyme's buried active site. In the models of soluble CYP2C9, the amplitude of these motions was higher when the FG loop was extended. The largest opening-closing motions were observed in the simulation of the product-bound 1R9O2 model and were attributed mainly to an increased flexibility of the BC loop (Fig. S6D), whereas the substrate-bound 1R9O2 was the least flexible of the structures with an extended FG loop. Moreover, the product-bound form was the most flexible structure among the soluble models with a helical FG loop. In the simulation of this model, the F' and G' helices unfolded and the G helix moved away from the BC loop leading to a significant increase of the root mean square deviation of the FG loop (Fig. S6E).

In the simulations of the membrane-bound models, the ligand-free structures were the most flexible, independent of the FG loop conformation. The stability of the F' and G' helices in the FG loop was not affected by the interaction with the lipid bilayer. In fact, the F' and G' helices were less stable in the simulations of soluble models of CYP2C9. The association with the membrane destabilized the interactions between L102 (BC loop) and I213 (F' helix) and I223 (G' helix) by favoring a motion of L102 away from this hydrophobic cluster. However, this did not result in a loss of stability due to new interactions formed by L102 with L234 and L208 which were not observed in the soluble models. Residues I215 (F' helix) and I222 (G' helix) interacted with the lipid tails. In the simulations of the soluble models with an extended FG loop, L102 interacted with I222 and I223, whereas in the simulations of the membrane-bound models, these interactions were not stable and L102 interacted either with the lipid tails or with L208 and L233 whereas I213, I215, and I222 were anchored in the membrane. I223 was oriented towards the active site independent of the FG loop conformation.

The flexibility of the BC loop observed in the simulations of both soluble and membrane-bound models resulted from the instability of the following interactions: (i) hydrophobic interactions between L102 and A103 and the FG loop, (ii) transient hydrogen bonds between R105 and E104 and H230, N231 (G helix) and D224 (FG loop, only in models with F' and G' helices), (iii) hydrophobic interactions between A106 and L234 and V237 (G helix), and (iv) hydrogen bonds between the backbone of A106 and N107 and/or the side chain of N207 and K241 (G helix) and E285 (I helix). Flexibility in the β1 sheet was a result of reorientation of F69 from the active site cavity to the protein surface. The dynamics of the interactions determining the positions of the BC loop and the β1 relative to the FG loop were similar in the simulations of soluble and membrane-bound models, except for those between L102 and the FG loop. The higher flexibility of the BC loop in the simulation of the product-bound 1R9O2 model was due to the loss of the hydrogen bonds between K241 and the BC loop and of the hydrophobic interactions involving A106.

### The opening and closing motions of putative substrate access and product release tunnels are correlated

**Figure 5:**
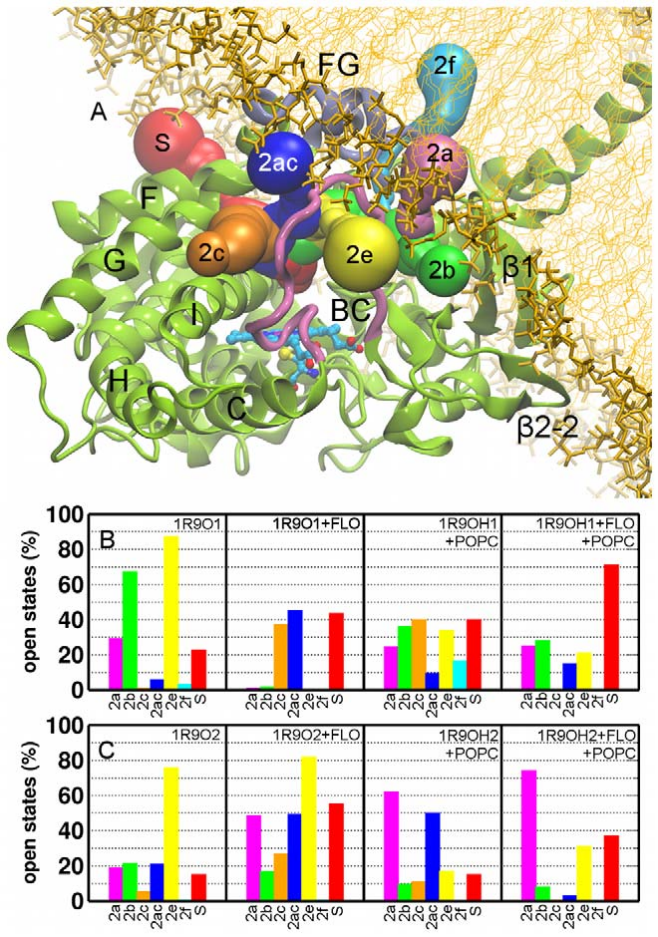
Tunnels from the CYP2C9 buried active site to the protein surface. (A) Cartoon representation of all tunnels that were observed to open during the atomic-resolution simulations, labeled according to the nomenclature of Cojocaru et al. [20]. The percentage of trajectory frames in which different channels were open (the smallest radius along the tunnel >1.2 Å) is shown for the models 1R9O1, 1R9OH1 (B), and 1R9O2, 1R9OH2 (C). The colors of the bars correspond to the colors assigned to each tunnel in (A).

**Figure 6:**
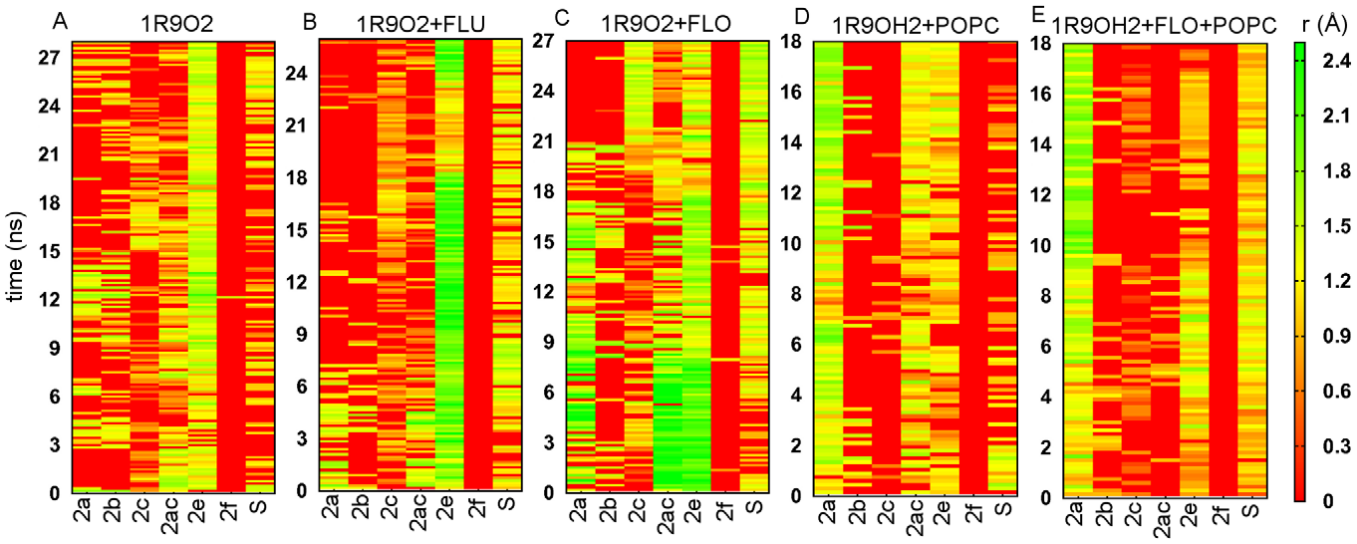
Correlation between the opening and closing of different tunnels during the simulations. The smallest radius along each tunnel is plotted during the atomic-resolution simulations of membrane-bound (1R9OH2) and soluble (1R9O2) CYP2C9. (A) The apo form of 1R9O2. (B) 1R9O2 with bound substrate (flurbiprofen (FLU)). (C) 1R9O2 with bound product (4-hydroxy-flurbiprofen (FLO)). (D) The apo form of 1R9OH2. (E) 1R9OH2 with bound product (FLO). The color map ranges from closed (red to dark yellow) to open tunnels (light yellow to green).

We investigated the opening and closing motions of the tunnels 2a, 2b, 2c, 2ac, 2e, 2f, and the solvent (S) tunnel (the nomenclature used is from [20]) (Fig. 5A). Each of these was found open in at least one snapshot of the simulations. In addition, these tunnels were identified as ligand exit routes in a separate set of simulations of soluble CYP2C9 models (unpublished data). The entrances of the tunnels were defined as described in Table 3 (see also Fig. S7). The fraction of open states of each tunnel in each simulation was computed (Fig. 5B,C). We identified three factors that influence the opening and closing motions in CYP2C9: (i) the conformation of the FG loop, (ii) ligand binding, and (iii) association with the membrane. Tunnel 2a is significantly more open in the models with an extended FG loop. The F' and G' helices restrict the opening of 2a, while favoring the opening of 2b which is mostly closed when the FG loop is extended. Tunnels 2c and S tended to be open more in the simulations of the product-bound models. This tendency was independent of the conformation of the FG loop and the association with the membrane in the case of S whereas for 2c it was evident only in the simulations of the soluble models. 2c also opened in the shorter simulation (with γ = 50 dyn/cm) of the apo form of the membrane-bound 1R9OH1 model. The interaction with membrane favored the opening of S in the models with a helical FG loop and restricted it in the other models. Remarkably, the opening of 2c and S correlates very well with the closing of 2a and/or 2b (Fig. 6 and S8). In particular, we observed the closing of 2a and opening of 2c after about 18–20 ns in the simulation of the product-bound, soluble 1R9O2 model (Fig. 6C). Moreover, this correlation was observed when comparing the simulations of the product-bound and ligand-free structures of the 1R9O1 model (Fig. S8A,B). The lipid bilayer favored the opening of 2a regardless of the conformation of the FG loop and or whether a ligand was bound. However, the amplitude of the opening of 2a was larger in the models with an extended conformation of the FG loop. In the simulations of these models, a POPC molecule was observed to penetrate into 2a with its choline methyl groups interacting with the protein residues L208, F69, F100, and W212. The opening of 2e was in general restricted by the presence of the lipids except that it was also closed in the simulation of the soluble, product-bound 1R9O1 model. Tunnel 2f was mostly closed in all the simulations, while opening of 2ac appeared to correlate either with the opening of 2a in the models with an extended FG loop (Fig. 6C,D) or with the opening of 2c in the models with F' and G' helices (Fig. S8B).

**Table 3:** Tunnels in CYP2C9.

Tunnel^1)^	Residues defining entrance (1R9O1)^2)^	Residues defining entrance (1R9O2)^2)^	Lining secondary structure^3)^
2a	F69, K72, P101	K72, P101, S220, P221	β1 sheet, FG, BC loops
2b	K72, I74, R97, I99	K72, I74, R97, I99	β1, β2 sheets, BC loop
2c	A106, R108, V237, E288	A106, R108, V237, E288	G, I helices, BC loop
2ac	P101, A106, P221, H230	L102, A106, I222, H230	G helix, BC loop
2e	G98, P101, A106, G109	G98, P101, A106, G109	BC loop
2f	P37, I45, N218, P221	I42, I47, I215, P211	FG loop, N-terminal coil
S (solvent)	C206, E300, R307, F476	C206, E300, R307, F476	F, E helices, β4 sheet

1. Nomenclature as in [20].

2. The tunnel entrance was defined as the geometric center of the Cα atoms of the residues listed.

3. The original secondary structure nomenclature defined for the bacterial P450s [26] was adapted to CYP2C9.

The opening of 2a, as well as the closing of 2e, was correlated with the opening of the internal aromatic gate formed by F100, F114, and F476. In addition, opening of 2a was facilitated by the loosening of the cluster of hydrophobic interactions around L102. 2c opened when the hydrogen bonds between K241 (G helix) and the backbone of the BC loop and the hydrophobic interactions between A106 (BC loop) and L234 and V237 (G helix) were disrupted. As in all other simulations, R108, which interacts directly with the substrate, kept its orientation towards the inside of the active site and the hydrogen bonding pattern between R108, N289, and D293 was maintained.

### The membrane stabilizes open conformations of the internal aromatic gate

**Figure 7:**
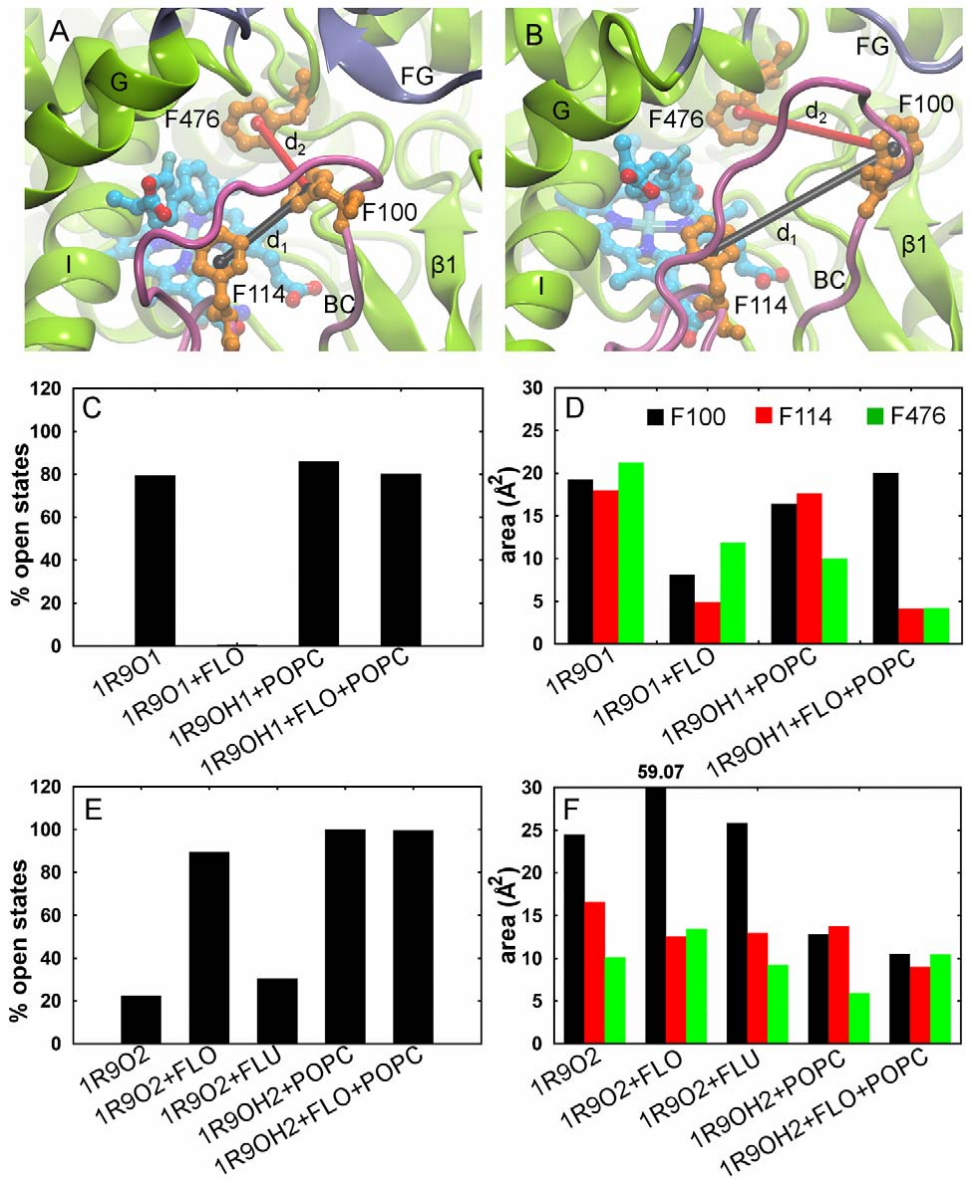
Conformational states of the internal aromatic gate formed by F100, F114 and F476. (A) Closed. (B) Open. The distances between the centers of the phenyl rings (d_1_: F100–F114 (black), d_2_: F100–F476 (red)) define the conformational state of the gate. The gate was considered open if d_2_>7 Å while d_1_ was used to define the position of F100 with respect to the other phenylalanines. (C, E) The percentages of trajectory frames in which the gate is open in the atomic-resolution simulations of models 1R9O1, 1R9OH1 (C) and 1R9O2, 1R9OH2 (E). (D, F) The area covered by the centers of the phenyl rings projected on the heme plane (see Fig. S10 for details) in the atomic-resolution simulations of models 1R9O1, 1R9OH1 (D) and 1R9O2, 1R9OH2 (F).

In the 1R9O crystal structure, the aromatic gate is closed and locks the substrate in the bound position above the heme center [25]. In the simulations, dynamics of the gate regulate tunnel opening motions. To calculate the percentage of each trajectory in which the gate is open, we monitored defined distances between the phenyl rings (Fig. 7A,B). We found that the association with the membrane stabilized an open conformation of the gate independent of the conformation of the FG loop (Fig. 7C,E). F100 adopted two positions when the gate was open, either close to F114 blocking the entrance of 2e (observed mostly in simulations of models with a helical FG loop) or close to F69 lining the entrance of 2a (observed mostly in simulations of models with an extended FG loop).

The amplitude of the gating motions was estimated by projecting the center of the phenyl rings on the heme plane and monitoring the area covered during the simulations: the greater the area, the more mobile the phenyl ring (Fig. S10). We found that in the simulations of the models with a helical FG loop, the motion of F114 was restricted by the bound product, the motion of F476 was restricted both by the bound product and by the interaction with the membrane, and the motion of F100 was not affected (Fig. 7D). These observations agree well with the position of F476 near the head group region of the lipid in these models, but not with the position of F100 in the simulation of the product-bound 1R9OH1 model (Fig. S9A,B). In the simulations of the models with an extended FG loop, the interaction with the membrane restricted the motion of F100 (Fig. 7E), while the motions of F114 and F476 were not affected. This is consistent with the position of F100 in the proximity of the lipid head groups in these models (Fig. S9C,D).

## Discussion

We have described a procedure to derive models of a CYP-membrane bilayer complex in an unbiased fashion that, unlike previous approaches to model CYP-membrane complexes, does not rely on input of any experimental data on the CYP-membrane interactions. From multiple coarse grained molecular simulations started with the globular domain in random orientations above or loosely inserted in the membrane and the statistical analysis of defined parameters, two predominant orientations of CYP2C9 in the membrane were found that were both equally consistent with available experimental data. These orientations differed in the conformation of the FG loop. The two protein-membrane configurations generated from coarse grained simulations were conserved in atomic-resolution simulations with only minor adjustments in protein orientation.

The main difficulty in the overall procedure for the deriving the CYP-membrane models is the generation of the initial system for the coarse grained simulations. We found that the pre-assembly of the lipid bilayer and the pre-simulation of the transmembrane helix inserted in the membrane were required. In test simulations of self-assembly [28],[29] of CYP2C9-membrane complexes, the N-terminal helix did not assembled in a transmembrane configuration (data not shown). The coarse grained force field does not maintain protein tertiary structure unless it is complemented by the application of an elastic network model to connect the C_α_ atoms [30], [31]. Thus the region restrained by the elastic network must be chosen carefully. In initial simulations with all residues observed in the crystal structure of CYP2C9 included in the elastic network model, the transmembrane configuration of the N-terminal helix of CYP2C9 was not stable (data not shown). Hence, we detached the unstructured region of 14 residues adjacent to the transmembrane helix from the elastic network and generated initial configurations by arbitrary modification of the backbone parameters in this region. It is difficult to achieve a full sampling of the conformations of this linker and, given the limitations of the coarse grained force field, to ensure that the conformations are plausible. Therefore, it is possible that the linker is trapped in a suboptimal local minimum after the atomic-resolution simulations. This limitation might be overcome in future applications of this procedure to generate protein-membrane complexes for other proteins with single helix membrane anchors by improvements in coarse grained force fields and the application of an iterative approach to gradually detach residues from the elastic network.

We found that the CYP2C9-membrane complexes were largely consistent with experiments. However, the comparison was limited by inconsistencies among different experiments and the diversity of CYP isoforms used in the experiments. The three main inconsistencies between our simulations and the experiments were: (i) the β1 sheet was inserted in the lipid bilayer despite microsomes being recognized by a site-specific antibody against the corresponding region in CYP2B1 [8], (ii) the coil region between the B helix and the BC loop was accessible to solvent despite experimental evidence for the association with membrane of the corresponding region in CYP2B1 [8], (iii) L380 was found in the region of the lipid head groups in the simulations whereas it was inferred to insert deepest in the membrane from tryptophan fluorescence quenching experiments of CYP2C2 [12]. Major reorientation of the protein would be required to improve the consistency with these experiments. However, this would diminish the consistency with other experiments and would impair the interaction with the CPR. A putative orientation with the heme tilt angle at nearly 90°, similar to the configurations proposed based on the crystal structures of CYP2C5 and CYP2C8 [13], [14]. would be consistent with the proposed position of L380 as the deepest in the membrane [12]. However, we did not observe such a configuration in our simulations. Furthermore, when we placed the protein in the membrane in such an orientation, we found that the model was not stable in atomic-resolution simulations (data not shown). We cannot exclude that CYPs undergo large rearrangements in the membrane and adopt conformations that may show greater consistency with experiments, however it is apparent that, with the conformations sampled, it is not possible for a CYP2C9 orientation in the membrane to simultaneously satisfy all experimental data.

In the models derived here the surface proposed to interact with the CPR [32] was exposed to the solvent. We predict that both orientations of CYP2C9 in the membrane are favorable for electron transfer from the CPR. CPR undergoes a major conformational rearrangement to achieve a conformation suitable for electron transfer to CYP [33]. We anticipate that the amplitude of this conformational transition in CPR is larger, and therefore more energetically costly, when the CYP is more tilted with respect to the lipid bilayer (i.e. has a lower value of the heme tilt angle). This implies that the electron transfer between CPR and CYP might be more efficient when the CYP is less tilted with respect to the lipid bilayer. This proposal could be investigated by extending the simulation protocol described to include CPR in the models. We used a membrane with a size chosen to permit this extension.

CYP2C9 remained in closed or nearly closed conformations in the simulations. We did not observe open conformations similar to those reported in crystal structures of other CYPs [17], [19], [27] or in a recent molecular dynamics study of soluble CYP2C9 [34]. Nevertheless, we identified motions that lead to opening of discrete tunnels from the enzyme's buried active site. Remarkably, we found that the opening and closing of the different tunnels were correlated and depended not only on the association with the lipid bilayer but also on ligand-binding and the conformation of the FG loop.

The tunnels 2c and S are accessible from the cytosol and opened preferentially in product-bound models of CYP2C9, suggesting that they are the preferred release tunnels for soluble products from CYP2C9. Both have been proposed to function as ligand exit routes in other CYPs [6], [21], [22]. The opening of tunnel 2a leading into the membrane was facilitated by the interaction of CYP2C9 with the bilayer and by the extended conformation of the FG loop. A role for this tunnel in mammalian CYPs has not been defined. We propose that 2a is the preferred substrate access tunnel from the lipid bilayer for liposoluble compounds. This proposal is supported by the correlation observed between the opening of 2a and the closing of 2c and S. 2a was previously described as the main ligand egress tunnel in three bacterial P450s which are soluble enzymes [35]. Additional evidence for substrate access from the membrane is provided by Berka et al. (unpublished data) who show that the preferred position of ibuprofen, a drug metabolized by CYP2C9, is near the lipid tails. Moreover, a charge-modifying sequence variation between CYP2C9 and CYP2C19, a closely related CYP that does not have the specificity for acidic compounds that CYP2C9 does, is found around the entrance of 2a [36]. Despite this evidence for selective roles of different tunnels in CYP2C9, we cannot exclude that each tunnel is used both for substrate access and product release. Tunnel 2b appears to be an alternative for 2a in the CYP2C9 models with a helical FG loop. The opening of 2b was facilitated by the F' helix blocking the entrance of 2a. However, 2b did not lead into the membrane and it remained closed whenever a larger opening of 2a was observed. 2b was also found closed in the structure of CYP2B4 in which 2a was wide open [17]. Therefore, we suggest that in the ER membrane, the opening of 2a is sufficiently large to permit ligand passage and therefore 2b does not have an obvious function. We found that 2f, an alternative tunnel pointing into the membrane, was closed in the simulations. In the case of a wider opening of CYP2C9, 2f could merge with 2a to form a large access channel from the lipid bilayer. From the wealth of data on substrate access and product release in mammalian CYPs, it becomes apparent that there are multiple routes which are used by different CYPs depending on the ligand properties. If proven, this hypothesis may provide new strategies for the inhibition of CYPs.

Mechanistically, we found that the opening of 2a was facilitated by the opening of the internal aromatic gate formed by F100, F114, and F476. F114 and F476 were showed to be required for the CYP2C9 activity, whereas mutations of F100 do not affect catalysis [37]. This finding apparently contradicts the evidence from the structure of the flurbiprofen-bound complex where F100 was found to lock the substrate in the catalytically productive position above the heme. In addition, we found extensive interactions between different ligands and F100 during ligand exit simulations of soluble models of CYP2C9 (unpublished data). The experiments were performed in the presence of dilauroylphosphatidylcholine (DLPC); therefore it is more appropriate to compare them with the simulations of the membrane-bound CYP2C9. We found that the membrane stabilizes open conformations of the aromatic gate with F100 further away from the heme center. Thus, we suggest that the closed state of the gate may not be crucial for the catalytic activity of CYP2C9 in the lipid bilayer.

In conclusion, we applied a procedure to model and simulate the membrane bound CYP2C9 that is not biased towards a certain orientation of the protein in the membrane. The protocol may be applied to model other mammalian CYPs, their interaction with CPR or, in general, to proteins anchored in the membrane by a single transmembrane helix. For CYP2C9, we show that the lipid bilayer may play an important role in the selection of different tunnels for ligand exchange between the environment and the enzyme's buried active site and suggest selective roles for different tunnels.

## Materials and Methods

### CYP2C9 models

We constructed 2 models of soluble CYP2C9 based on the flurbiprofen-bound crystal structure (pdbid 1R9O, 1.9 Å resolution) [25]: (i) 1R9O1 has 2 small helices in the FG loop (F' and G'), and (ii) 1R9O2 has a small β-sheet in the mostly unstructured FG loop and is similar to an unpublished crystal structure of CYP2C9 (Eric Johnson, personal communication). Details of the model building procedure are given in the Supporting Information (Protocol S1).

The procedure used to model the membrane-bound CYP2C9 is outlined in Fig. 1. We predicted (using PSIPRED [38] and TMHMM [39]) that residues 1 to 22 adopt a helical conformation and built the corresponding ideal α-helix with MODELLER [40]. Residues 23 to 25 connecting the last residue of the N-terminal transmembrane helix and the first residue in the crystal structure were modelled in a random-coil conformation. The 25-residue long N-terminal peptide was then converted to a coarse grained representation (coarse grained particles were placed at the average positions of their corresponding non-hydrogen atoms) and inserted in a pre-equilibrated coarse grained model of a bilayer composed of 608 POPC molecules (Fig. 1, Step 1). The bilayer was chosen to be large enough to allow the insertion of CYP2C9 and the CPR. The POPC molecules that had particles within 3 Å of particles of the peptide were removed. The resulting N-terminal peptide-bilayer system was simulated for 3 µs (in a single simulation) as described below (Fig. 1, Step 2). The inclination of the transmembrane helix reached an equilibrium value of 12±6°.

Two complete coarse grained models of the membrane-bound CYP2C9 (1R9OH1 and 1R9OH2) were built by attaching the globular domain (models 1R9O1 and 1R9O2 pre-converted to a coarse grained representation) to the final structure of the simulated N-terminal peptide.

### Coarse grained simulations

The coarse grained parameters for POPC were taken from [41], and for the protein from [30], [42]. An elastic network model connecting the protein backbone particles closer than 7 Å with a spring constant k = 10.75 kcal*mol-1 Å-2 was applied to maintain the protein secondary and tertiary structure during the simulations [30], [31]. Coarse grained parameters for the heme group are not available. Therefore, we decided to remove the heme from the protein during the coarse grained simulations. Because the heme is buried in the protein, it will not affect the CYP-membrane interaction and its role in the maintenance of the protein tertiary structure is mimicked by the elastic network model. The simulations were performed with the GROMACS 4 program [43] under periodic boundary conditions, in coarse grained water, in the NPT ensemble using the Berendsen weak coupling algorithm [44] to maintain the temperature at 300 K and the pressure at 1 atm. The time step was 20 fs. The non-bonded (van der Waals and electrostatic) interactions were evaluated with a cutoff of 14 Å. The dielectric constant was 20. Semi-isotropic pressure scaling (with relaxation time τ_p_ = 2 ps in all three space directions) was used to account for fluctuations of the membrane size.

An initial set of 2 simulations, each of 3 µs, was performed with the membrane-bound 1R9OH1 and 1R9OH2 models during which the N-terminal helix adopted an orientation parallel to the head groups at the membrane surface (data not shown). The transmembrane configuration of the helix was stable only when residues 23 to 37 in the linker peptide were decoupled from the elastic network model, thus allowing different backbone configurations in the linker peptide. 7 1R9OH1 and 5 1R9OH2 structures with different orientations of the globular domain with respect to the bilayer were constructed by randomly modifying the dihedral angles in the linker peptide backbone (Fig. 1, Step 3). Each of these 12 structures was used as the starting structure for a 1 µs simulation during which snapshots were recorded every 0.4 ns (Fig. 1, Step 4). The protein orientation with respect to the lipid bilayer was evaluated by considering all the snapshots recorded in the 12 simulations except for the first 100 ns of each simulation.

### Conversion of coarse grained models to atomic resolution

The conversion protocol for the membrane was similar to that described by [45]. However, we implemented it in VMD [46] and adapted it to be compatible with the generalized amber force field (gaff) for POPC lipids [47] which we used in the atomic resolution simulations. The rmsd between the initial coarse-grained and the converted atomic resolution POPC molecules was less than 1.5 Å (Fig. S11), see Protocol S2 in the Supporting Information for details of the procedure and the files necessary for its application.

The globular domain (residues 37–492) of CYP2C9 was converted by superimposing the atomic resolution models 1R9O1 and 1R9O2 (taken directly after the model building procedure) on the selected coarse grained models of the membrane-bound CYP2C9. This ensured that the atomic resolution simulations of the membrane-bound and soluble CYP2C9 were started with the same initial models of the globular domain. The transmembrane helix (residues 1–22) was converted by superimposing the ideal α-helix on the selected coarse grained models. The linker peptide (residues 23–36) was converted with a simulated annealing protocol (Protocol S1) in which the positions of the Cα atoms were restrained to the positions of the coarse grained backbone particles (Fig. 1, Step 6).

### Atomic-resolution simulations

Atomic-resolution simulations (Fig. 1, Step 7) were performed with the following models: (i) 1R9O1, (ii) 1R9O1 + FLO, (iii) 1R9O2, (iv) 1R9O2 + FLU, (v) 1R9O2 + FLO, (vi) 1R9OH1 + POPC, (vii) 1R9OH1 + FLO + POPC, (viii) 1R9OH2 + POPC, (ix) 1R9OH2 + FLO + POPC. FLU  =  flurbiprofen (substrate) and FLO  = 4-hydroxy-flurbiprofen (product). The first 5 models are soluble truncates of CYP2C9 while the last 4 are atomic-resolution models of the membrane-bound CYP2C9. In the models of the apo form of the protein, 5 water molecules were placed in positions originally occupied by the ligand. The simulations were performed with NAMD [48] in explicit solvent in 150 mM NaCl under periodic boundary conditions. The Particle Mesh Ewald method was used to evaluate the electrostatic interactions. The “ff99sb” version of the AMBER force field [49] was used for the protein and gaff was used for the lipids [47]. The ion parameters were taken from [50]. The partial atomic charges for the ligands were derived using the procedure of restrained fit to the Hartree-Fock 6-31G* electrostatic potential (RESP) with the RED program. The heme parameters were provided by D. Harris with the partial atomic charges derived from DFT calculations [51]. The RATTLE algorithm was used to constrain the bonds to hydrogens. The temperature was controlled with Langevin dynamics (damping coefficient 1≤ γ_T_ ≤5 ps^−1^ during equilibration and γ_T_  = 0.5 ps^−1^ during production). Pressure was controlled with the Nose-Hoover Langevin Piston method (oscillation period ω  = 100 fs and damping coefficient γ_P_  = 50 fs^−1^ during equilibration and ω  = γ_P_ = 1000 fs during production). The time step was 1 fs during equilibration and 1.5 fs during production. The systems were equilibrated as described in the Supporting Information (Protocol S3) and then production runs were carried out as summarized in Table 1.

For snapshots at 150 ps intervals along each simulation, we calculated 10 tunnels with MOLE [52] and saved 200 segments and their radii along each tunnel. Each computed tunnel was assigned to one of the seven tunnels listed in Table 3 if at least one segment of the tunnel was less than 5 Å from the entrance of the tunnel and the entrances of the other six tunnels were further away from each segment of the tunnel. The assignments were accurate because the distance between any two tunnel entrances was larger than the maximum displacement of each entrance due to internal protein motions (Table S2). If the minimum radius of the segments along a tunnel was greater than 1.2 Å, the tunnel was considered open to permit the passage of at least 1 water molecule. This value was chosen to be slightly lower than the usual probe radius of water of 1.4 Å because the radii calculated with MOLE are smaller than the actual radii of the tunnels. The procedure was implemented in VMD [46].

## Supporting Information

Figure S1 Root mean square deviation (rmsd) compared to the initial structure computed for the coarse grain particles of the protein backbone of the globular domain of CYP2C9 during the coarse grained simulations. Histograms with the bin size 0.1 Å were calculated from: (A) the 7 coarse grained simulations of the 1R9OH1 model; (B) the 5 coarse grained simulations of the 1R9OH2 model. Two examples of the time evolution of the rmsd during a coarse grained simulation are shown, one for the 1R9OH1 model (C) and one for the 1R9OH2 model (D).
(TIFF)

Figure S2 Variance in the angles defining the protein orientation in the membrane due to internal protein dynamics. Histograms for the angles α (black) and β (red) were recalculated from all coarse-grained simulations of models 1R9OH1 (A) and 1R9OH2 (B) after superimposing the protein backbone (the particles corresponding to C_α_ atoms). In each trajectory, the last snapshot in which the three orientation parameters (d, α, and β) were within 3% of the peak values of the corresponding histograms in Fig. 3 (see main manuscript for details) was selected for superposition (reference snapshot). The angle variance was calculated by subtracting the value of the angle in the reference snapshot from the corresponding value in each snapshot of the trajectory.
(TIFF)

Figure S3 Root mean square deviation (rmsd) of the globular domain of CYP2C9 during the equilibration phase for the atomic resolution simulations. For each model, the structure after the initial energy minimization (before equilibration) was used as the reference. The rmsd was calculated for the backbone atoms of the globular domain (residues 47 to 492) after they were superimposed on the reference structure in trajectory snapshots recorded every 1 ps. The rmsd between the reference structure and the 1R9O crystal structure varied between 0.8 Å and 1.2 Å and was calculated by superimposing the backbone atoms of residues 47 to 200 and 250 to 492.
(TIFF)

Figure S4 Number of contacts between protein residues and POPC lipids. The distance between each protein residue and 3 different parts of the lipids, head (black), middle (red), tail (green) were calculated. A contact was recorded if any heavy atom of the protein residue was closer than 4 Å to any heavy atom of the lipid part. The protein residues were split in 6 categories: (i) hydrophobic (L, I, V), (ii) aromatic (F, Y, W), (iii) hydrophilic (S, T, N, Q), (iv) charged (D, E, R, K), (v) glycine, (vi) proline. (A) The lipid parts illustrated on a lipid molecule). (B,C) The number of protein-lipid contacts in (B) the product bound 1R9O1H model, and (C) the product bound 1R9O2H model. The number of H, C, M, and A residues in contact with the lipids was below 3 in each case, thus we did not include them in the analysis.
(TIFF)

Figure S5 Charged and hydrophobic protein residues in contact with lipids. The charged residues, R, K (dark blue), D, E (dark red) in contact with lipids are shown on the last snapshot of the simulations of the product bound 1R9O1H (A) and 1R9O2H (B) models. The hydrophobic residues L, I and V (cyan) in contact with lipids are shown on the last snapshot of the simulations of the product bound 1R9O1H (C) and 1R9O2H (D) models. The view is perpendicular to the membrane, along the transmembrane helix.
(TIFF)

Figure S6 Root mean square deviations of the globular domain, FG, and BC loops during the atomic resolution simulations. For each model, the reference structure was chosen as described in Figure S3. The rmsd was calculated for the backbone atoms of the globular domain (residues 47 to 492), the BC (residues 93 to 116), and the FG (residues 208 to 230) loops after they were superimposed on the reference structure in trajectory snapshots recorded every 1.5 ps.
(TIFF)

Figure S7 Definition of tunnel entrances. A set of C_α_ atoms lining each tunnel was chosen and the entrance was defined as the geometric center of these atoms. The entrances of the 2a, 2b, 2c, 2ac, and 2e tunnels in the 1R9O1 and 1R9O2 models (same as in 1R9O1H and 1R9O2H) are shown in (A) and (C), while the entrances of tunnels 2f and S are shown in (B) and (D). The coloring of the tunnels follows the scheme in Fig. 5 (see main manuscript). The C_α_ atoms are labeled with amino-acid type and number and are shown as cyan spheres marked with dots colored by the tunnel(s) they define (some Cα atoms define more than one tunnel entrance, and thus are marked with more than one dot).
(TIFF)

Figure S8 Correlation between the opening and closing of different tunnels in CYP2C9. The analysis of the atomic resolution simulations of the models with a helical FG loop is shown: (A) the apo form of 1R9O1. (B) the product-bound form of 1R9O1. (C) the apo form of the membrane-bound 1R9OH1. (D) the product-bound form of the membrane bound 1R9OH1. (see also Fig. 6)
(TIFF)

Figure S9 Distances between the residues of the internal aromatic gate and the lipid bilayer. Histograms of the distances between the phenyl ring centers of F100, F114, and F476 and the average center of the lipid head groups projected on the z axis are shown. (A) 1R9O1H. (B) 1R9O1H+FLO. (C) 1R9O2H. (D) 1R9O2H+FLO. The reference (corresponding to the null distance, marked with thick black lines) was chosen as the calculated average of the z coordinates of the NH_4_^+^ group of the POPC lipids. A negative distance means that the phenyl rings are below the reference, closer to the lipid bilayer.
(TIFF)

Figure S10 Procedure to estimate the motion of the internal aromatic gate. (A) Illustration of the procedure to calculate the area covered by the motion of phenylalanine side-chains in the heme plane. The heme plane was defined by two vectors (v_1_ and v_2_) that connect the atoms NA with NC and NB with ND in the porphyrin ring of the heme. The heme plane was translated and rotated to coincide with the xy plane (the z coordinate of each projection point was 0). The position of the phenyl ring centers of F100, F114, and F476 was projected on the heme plane at every time frame of the simulation. The motion of the phenyl rings in the simulation is illustrated with arrows. (B) The areas covered by the phenyl ring centers in the heme plane (shown as black, red, and green surfaces) were estimated as the difference between the integrals of the “minimum” and “maximum” graphs of each surface (blue and violet lines). To obtain these graphs, the range of the x coordinate was split in bins of 0.2 Å and the minimum and maximum values of the y coordinate were calculated in each bin. The latter step was iterated 3 times, after each time the calculated minimum and maximum y values were discarded. As a result, the outliers at the edges of the surfaces were discarded.
(TIFF)

Figure S11 Procedure to convert the coarse-grained POPC molecules to atomic resolution. Histograms for the rmsd between the coarse-grained and the converted models at atomic resolution for all POPC molecules were calculated for the head group (black), the long (red) and the short (green) lipid tails before the final energy minimization step (right after the conversion) and for all lipids (blue) after the final energy minimization.
(TIFF)

Table S1 Contacts between CYP2C9 models and the lipid bilayer
(DOC)

Table S2 Positional variance of the tunnel entrances. Table S2 A: Displacement of tunnel entrances. Table S2 B: Distances between tunnel entrances
(DOC)

Protocol S1 Procedure to construct the models of the soluble CYP2C9.
(DOC)

Protocol S2 Procedure to convert coarse-grained to atomic resolution POPC molecules. Protocol_S2.tar.bz2 contains the files necessary for carrying out this procedure: (i) a full atomic-resolution library of POPC lipids containing 1454 different conformations, (ii) the force field parameters to be used together with the library (with permission from Martinek T. and Jojart B.), (iii) the conversion program written in tcl for use with the VMD program, (iv) a bash script that uses the AMBER software to perform the final minimization step and assembles the converted membrane.
(BZ2)

Protocol S3 Procedure to equilibrate the atomic-resolution models.
(DOC)
