# Supplementary figures and images for: Structure and Dynamics of the Membrane-Bound Cytochrome P450 2C9
Source: PLoS Comput Biol. 2011 Aug 11;7(8):e1002152. doi: 10.1371/journal.pcbi.1002152 (PMC3154944; doi:10.1371/journal.pcbi.1002152)

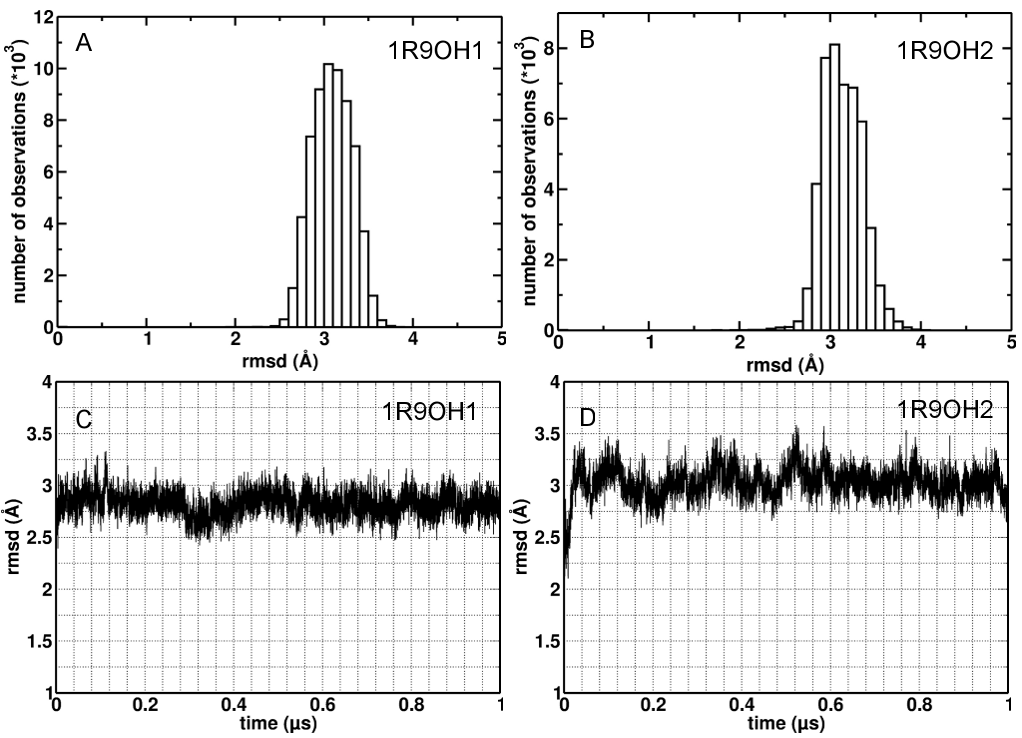

Supplement: Figure S1 — Root mean square deviation (rmsd) compared to the initial structure computed for the coarse grain particles of the protein backbone of the globular domain of CYP2C9 during the coarse grained simulations. Histograms with the bin size 0.1 Å were calculated from: (A) the 7 coarse grained simulations of the 1R9OH1 model; (B) the 5 coarse grained simulations of the 1R9OH2 model. Two examples of the time evolution of the rmsd during a coarse grained simulation are shown, one for the 1R9OH1 model (C) and one for the 1R9OH2 model (D). (TIFF) [file pcbi.1002152.s001.tiff]

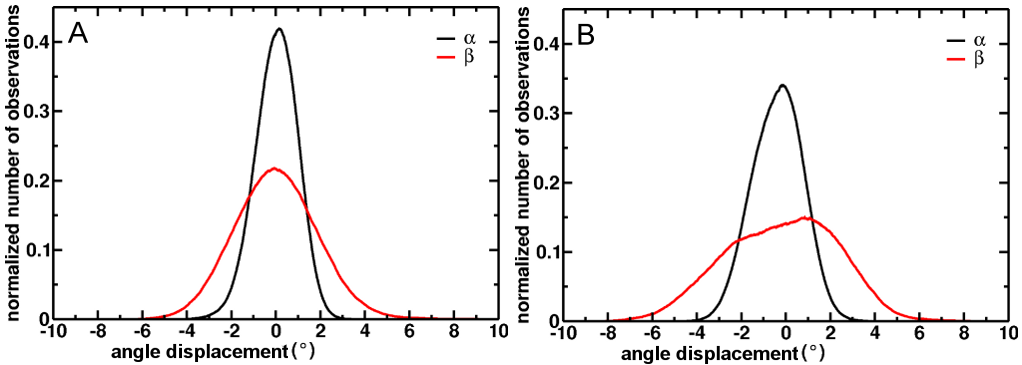

Supplement: Figure S2 — Variance in the angles defining the protein orientation in the membrane due to internal protein dynamics. Histograms for the angles α (black) and β (red) were recalculated from all coarse-grained simulations of models 1R9OH1 (A) and 1R9OH2 (B) after superimposing the protein backbone (the particles corresponding to Cα atoms). In each trajectory, the last snapshot in which the three orientation parameters (d, α, and β) were within 3% of the peak values of the corresponding histograms in Fig. 3 (see main manuscript for details) was selected for superposition (reference snapshot). The angle variance was calculated by subtracting the value of the angle in the reference snapshot from the corresponding value in each snapshot of the trajectory. (TIFF) [file pcbi.1002152.s002.tiff]

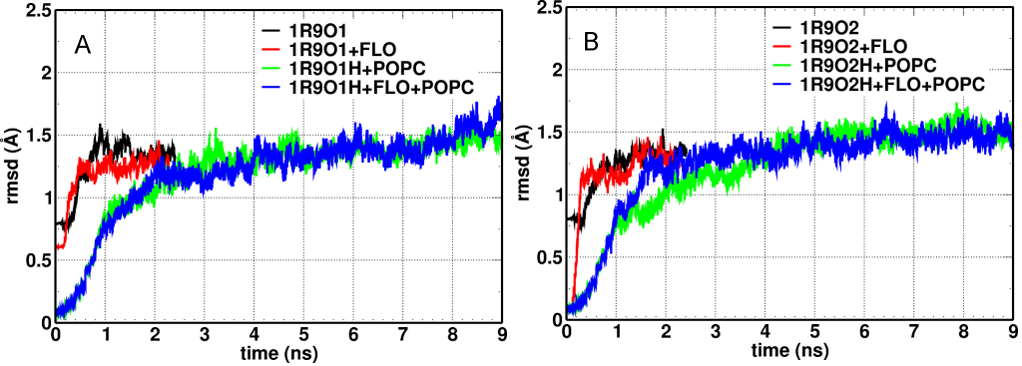

Supplement: Figure S3 — Root mean square deviation (rmsd) of the globular domain of CYP2C9 during the equilibration phase for the atomic resolution simulations. For each model, the structure after the initial energy minimization (before equilibration) was used as the reference. The rmsd was calculated for the backbone atoms of the globular domain (residues 47 to 492) after they were superimposed on the reference structure in trajectory snapshots recorded every 1 ps. The rmsd between the reference structure and the 1R9O crystal structure varied between 0.8 Å and 1.2 Å and was calculated by superimposing the backbone atoms of residues 47 to 200 and 250 to 492. (TIFF) [file pcbi.1002152.s003.tiff]

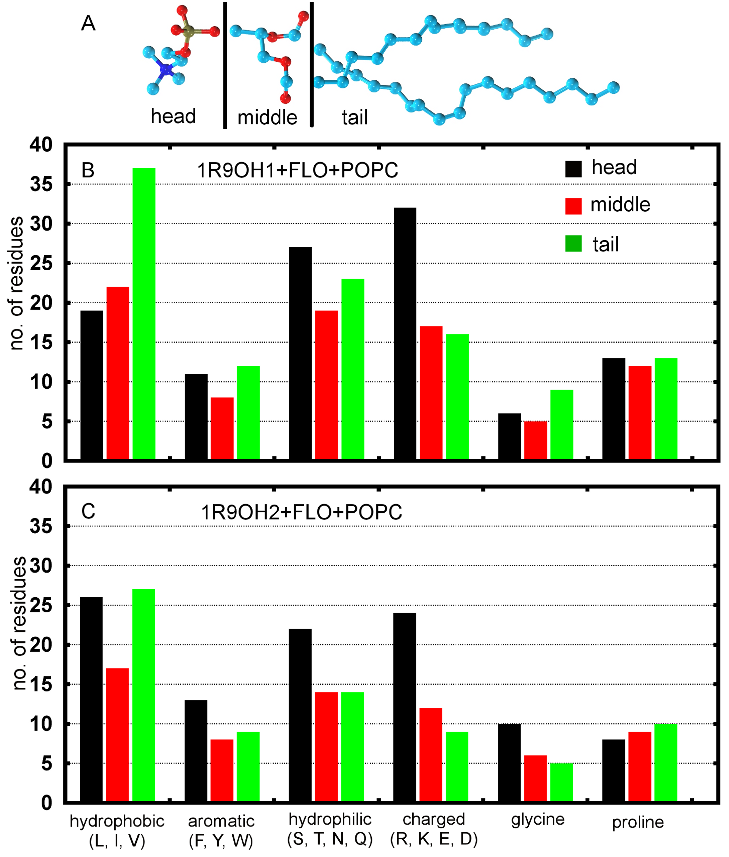

Supplement: Figure S4 — Number of contacts between protein residues and POPC lipids. The distance between each protein residue and 3 different parts of the lipids, head (black), middle (red), tail (green) were calculated. A contact was recorded if any heavy atom of the protein residue was closer than 4 Å to any heavy atom of the lipid part. The protein residues were split in 6 categories: (i) hydrophobic (L, I, V), (ii) aromatic (F, Y, W), (iii) hydrophilic (S, T, N, Q), (iv) charged (D, E, R, K), (v) glycine, (vi) proline. (A) The lipid parts illustrated on a lipid molecule). (B,C) The number of protein-lipid contacts in (B) the product bound 1R9O1H model, and (C) the product bound 1R9O2H model. The number of H, C, M, and A residues in contact with the lipids was below 3 in each case, thus we did not include them in the analysis. (TIFF) [file pcbi.1002152.s004.tiff]

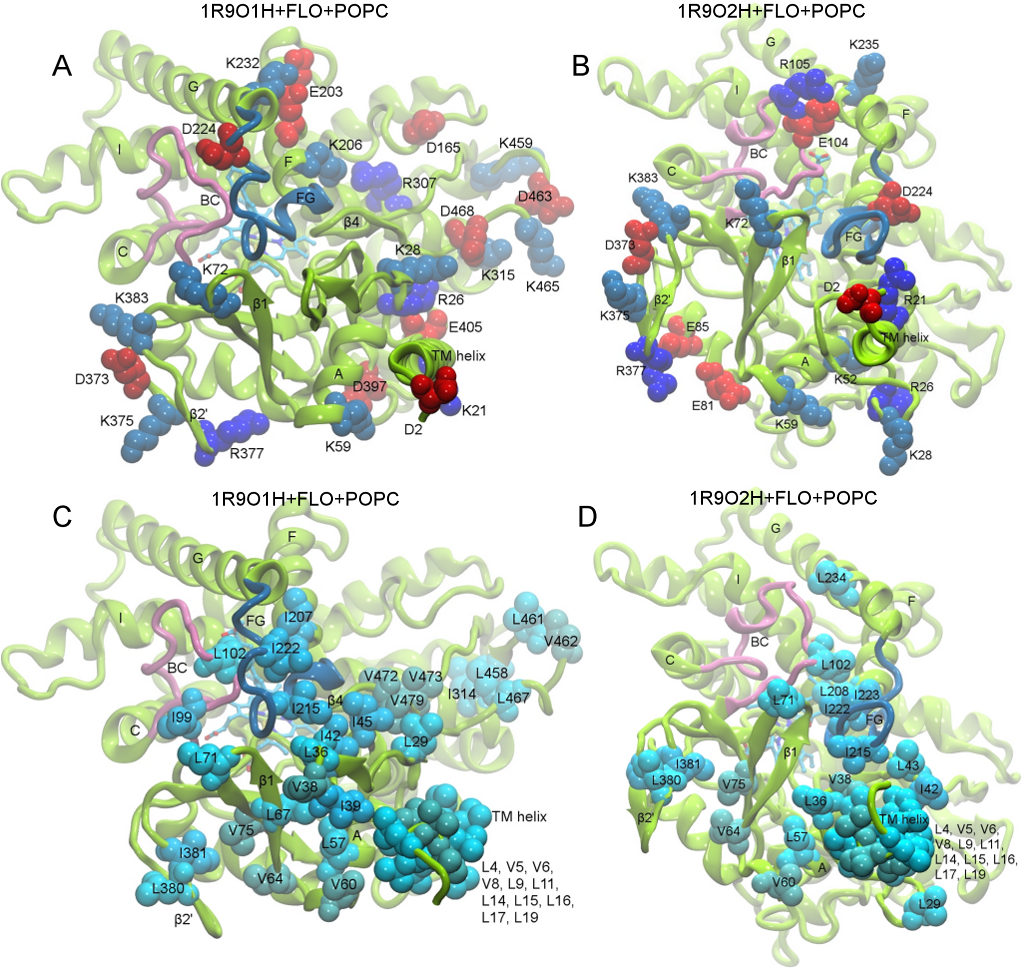

Supplement: Figure S5 — Charged and hydrophobic protein residues in contact with lipids. The charged residues, R, K (dark blue), D, E (dark red) in contact with lipids are shown on the last snapshot of the simulations of the product bound 1R9O1H (A) and 1R9O2H (B) models. The hydrophobic residues L, I and V (cyan) in contact with lipids are shown on the last snapshot of the simulations of the product bound 1R9O1H (C) and 1R9O2H (D) models. The view is perpendicular to the membrane, along the transmembrane helix. (TIFF) [file pcbi.1002152.s005.tiff]

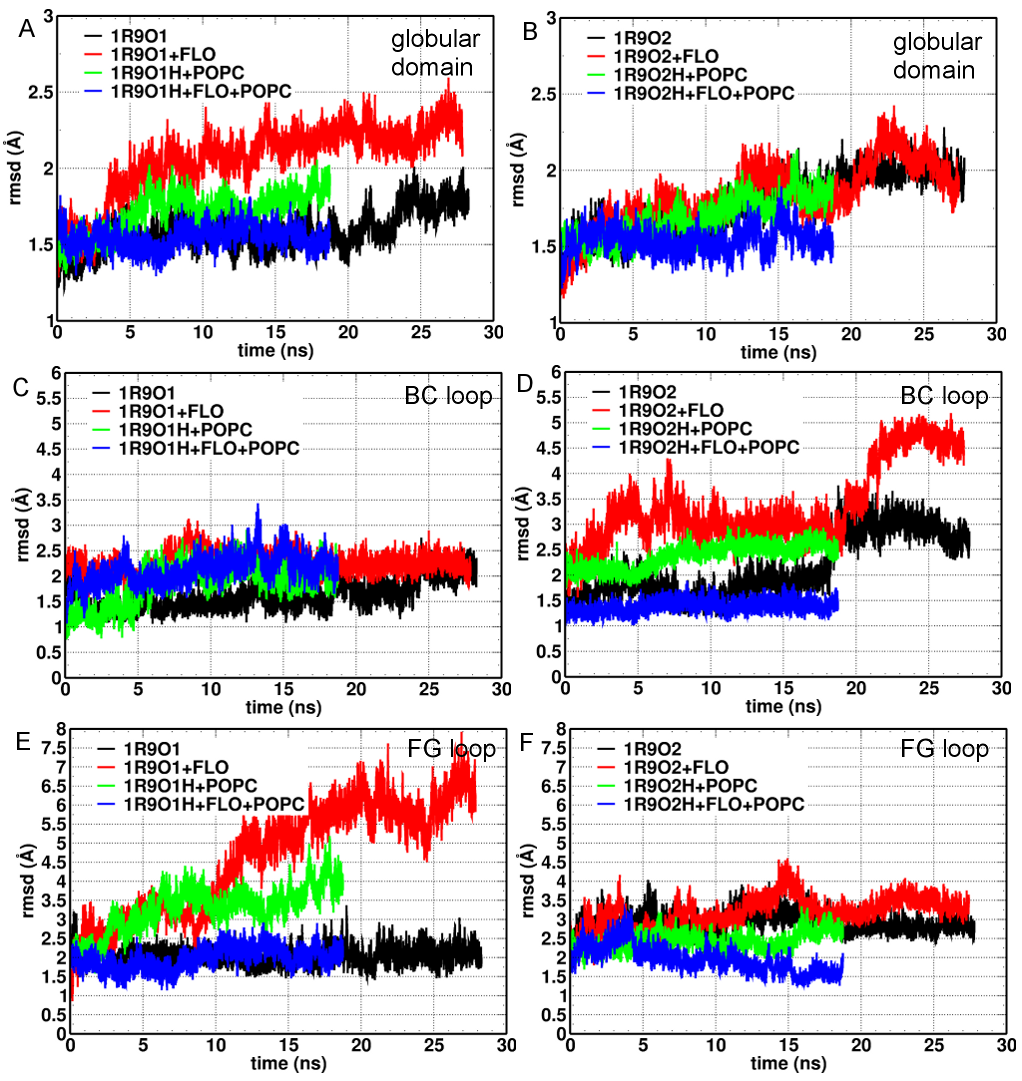

Supplement: Figure S6 — Root mean square deviations of the globular domain, FG, and BC loops during the atomic resolution simulations. For each model, the reference structure was chosen as described in Figure S3. The rmsd was calculated for the backbone atoms of the globular domain (residues 47 to 492), the BC (residues 93 to 116), and the FG (residues 208 to 230) loops after they were superimposed on the reference structure in trajectory snapshots recorded every 1.5 ps. (TIFF) [file pcbi.1002152.s006.tiff]

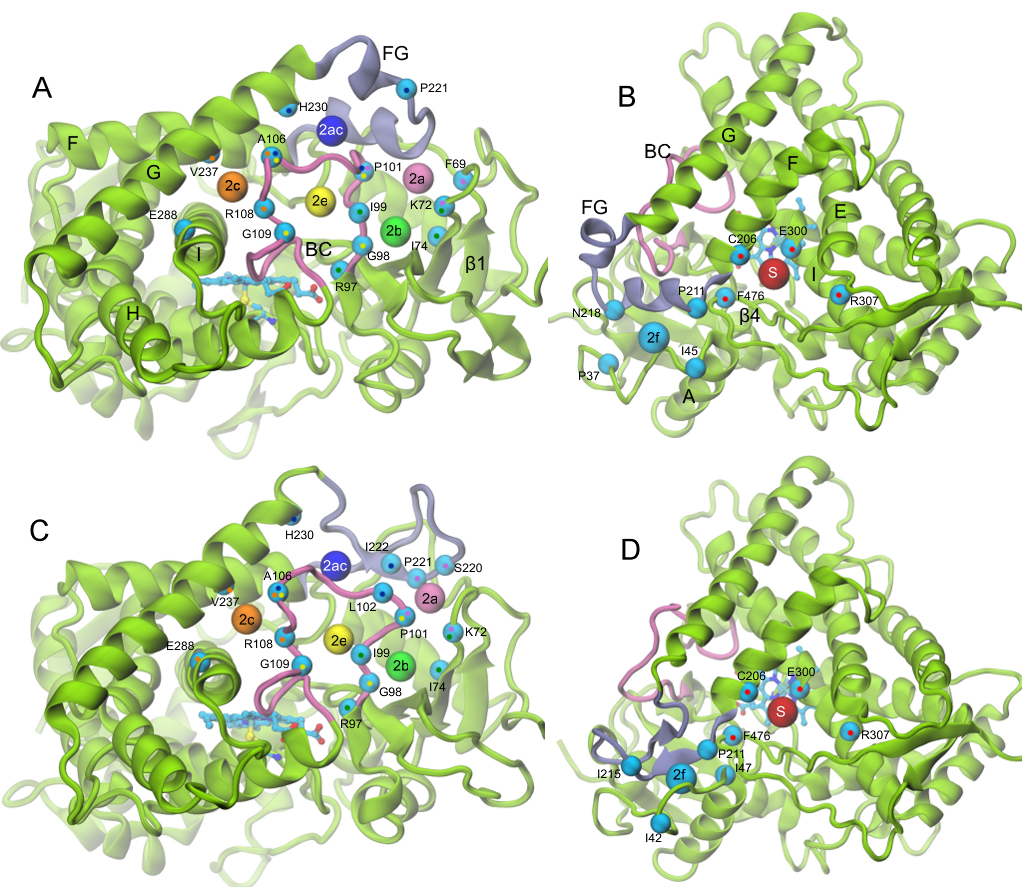

Supplement: Figure S7 — Definition of tunnel entrances. A set of Cα atoms lining each tunnel was chosen and the entrance was defined as the geometric center of these atoms. The entrances of the 2a, 2b, 2c, 2ac, and 2e tunnels in the 1R9O1 and 1R9O2 models (same as in 1R9O1H and 1R9O2H) are shown in (A) and (C), while the entrances of tunnels 2f and S are shown in (B) and (D). The coloring of the tunnels follows the scheme in Fig. 5 (see main manuscript). The Cα atoms are labeled with amino-acid type and number and are shown as cyan spheres marked with dots colored by the tunnel(s) they define (some Cα atoms define more than one tunnel entrance, and thus are marked with more than one dot). (TIFF) [file pcbi.1002152.s007.tiff]

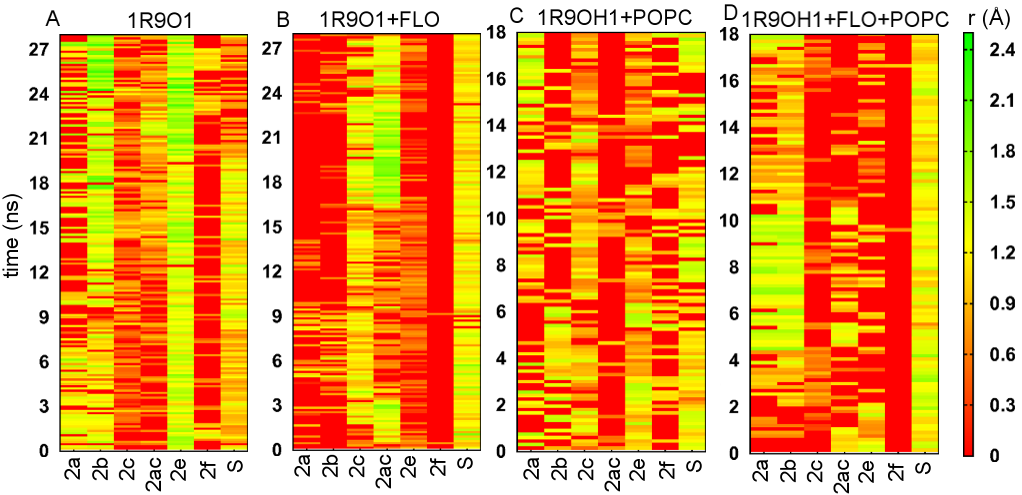

Supplement: Figure S8 — Correlation between the opening and closing of different tunnels in CYP2C9. The analysis of the atomic resolution simulations of the models with a helical FG loop is shown: (A) the apo form of 1R9O1. (B) the product-bound form of 1R9O1. (C) the apo form of the membrane-bound 1R9OH1. (D) the product-bound form of the membrane bound 1R9OH1. (see also Fig. 6) (TIFF) [file pcbi.1002152.s008.tiff]

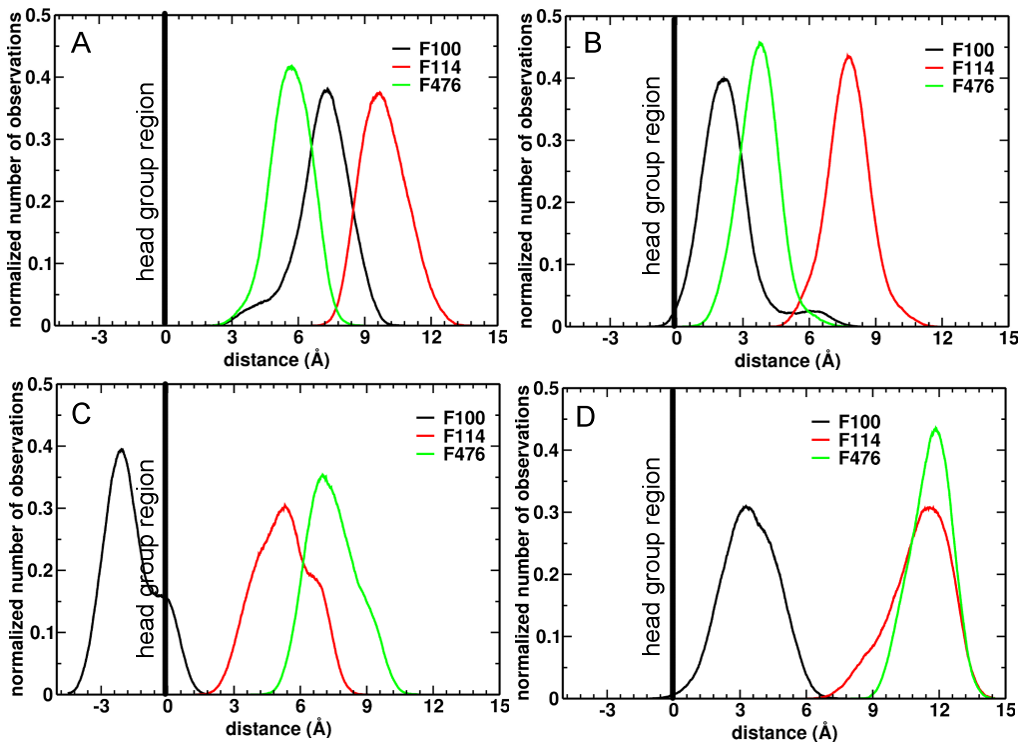

Supplement: Figure S9 — Distances between the residues of the internal aromatic gate and the lipid bilayer. Histograms of the distances between the phenyl ring centers of F100, F114, and F476 and the average center of the lipid head groups projected on the z axis are shown. (A) 1R9O1H. (B) 1R9O1H+FLO. (C) 1R9O2H. (D) 1R9O2H+FLO. The reference (corresponding to the null distance, marked with thick black lines) was chosen as the calculated average of the z coordinates of the NH4 + group of the POPC lipids. A negative distance means that the phenyl rings are below the reference, closer to the lipid bilayer. (TIFF) [file pcbi.1002152.s009.tiff]

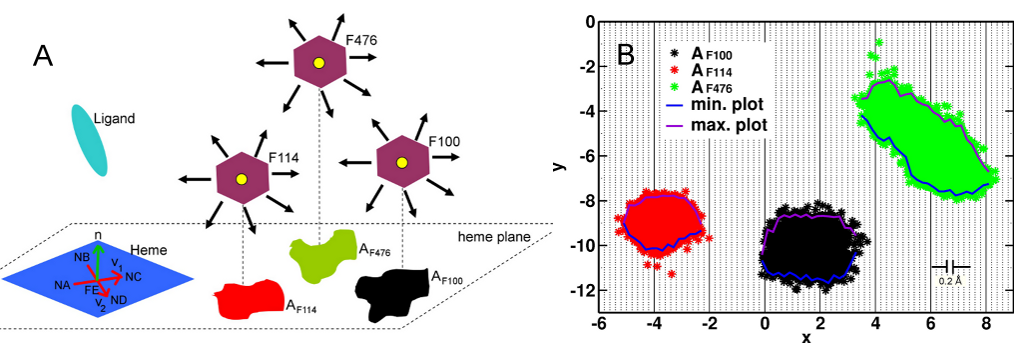

Supplement: Figure S10 — Procedure to estimate the motion of the internal aromatic gate. (A) Illustration of the procedure to calculate the area covered by the motion of phenylalanine side-chains in the heme plane. The heme plane was defined by two vectors (v1 and v2) that connect the atoms NA with NC and NB with ND in the porphyrin ring of the heme. The heme plane was translated and rotated to coincide with the xy plane (the z coordinate of each projection point was 0). The position of the phenyl ring centers of F100, F114, and F476 was projected on the heme plane at every time frame of the simulation. The motion of the phenyl rings in the simulation is illustrated with arrows. (B) The areas covered by the phenyl ring centers in the heme plane (shown as black, red, and green surfaces) were estimated as the difference between the integrals of the “minimum” and “maximum” graphs of each surface (blue and violet lines). To obtain these graphs, the range of the x coordinate was split in bins of 0.2 Å and the minimum and maximum values of the y coordinate were calculated in each bin. The latter step was iterated 3 times, after each time the calculated minimum and maximum y values were discarded. As a result, the outliers at the edges of the surfaces were discarded. (TIFF) [file pcbi.1002152.s010.tiff]

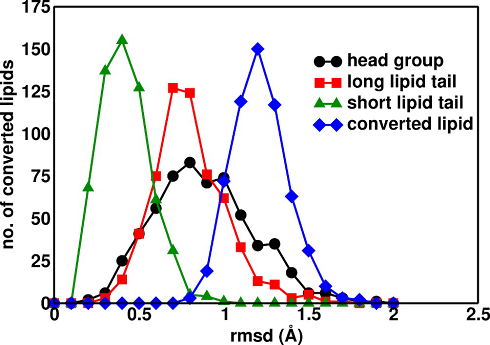

Supplement: Figure S11 — Procedure to convert the coarse-grained POPC molecules to atomic resolution. Histograms for the rmsd between the coarse-grained and the converted models at atomic resolution for all POPC molecules were calculated for the head group (black), the long (red) and the short (green) lipid tails before the final energy minimization step (right after the conversion) and for all lipids (blue) after the final energy minimization. (TIFF) [file pcbi.1002152.s011.tiff]
