# Supplementary material for: Structure and Dynamics of the Membrane-Bound Cytochrome P450 2C9
Source: PLoS Comput Biol. 2011 Aug 11;7(8):e1002152. doi: 10.1371/journal.pcbi.1002152 (PMC3154944; doi:10.1371/journal.pcbi.1002152)
Supplement: Table S1 — Contacts between CYP2C9 models and the lipid bilayer (DOC) [file pcbi.1002152.s012.doc]

**Table S1**: Contacts between CYP2C9 models and the lipid bilayer

| model | no. of contacts^*^ | % of catalytic domain in contact | secondary structure^**^ |
| --- | --- | --- | --- |
| 1R9OH1 | 157 (112) ± 9 | 31.9 | αA, β1, αB-αC, αD-αE,  αF-αG, αI-αJ, β3, αL-β4,  β4, β4-β5, β5, β5-Nt |
| 1R9OH1+FLO | 167 (124) ± 6 | 33.9 | αA, β1, αB-αC, αF-αG, β3, αL-β4,  β4, β4-β5, β5, β5-Nt |
| 1R9OH2 | 128 (85) ± 6 | 26.1 | αA, β1, αB, αB-αC,  αF-αG, β3, β4 |
| 1R9OH2+FLO | 122 (81) ± 5 | 24.8 | αA, β1, αB-αC,  αF-αG, αG, β3, β4 |

* The number of contacts between the globular domain (excluding the N-terminal trans-membrane helix and the linker) and the membrane is shown in round brackets.

** The secondary structure elements are labeled with “α” for α-helices and “β” for β-sheets and the numbering corresponds to the original nomenclature of the CYP fold (see main text).
