# Supplementary material for: Structure and Dynamics of the Membrane-Bound Cytochrome P450 2C9
Source: PLoS Comput Biol. 2011 Aug 11;7(8):e1002152. doi: 10.1371/journal.pcbi.1002152 (PMC3154944; doi:10.1371/journal.pcbi.1002152)
Supplement: Table S2 — Positional variance of the tunnel entrances. Table S2 A: Displacement of tunnel entrances. Table S2 B: Distances between tunnel entrances (DOC) [file pcbi.1002152.s013.doc]

**Table S2**: Positional variance of the tunnel entrances during the simulations

**Table S2A**: Displacement of tunnel entrances (Å)

| Tunnel | 1R9O1 | 1R9O2 | 1R9O1H | 1R9O2H |
| --- | --- | --- | --- | --- |
| 2a | 1.54 ± 0.39 | 1.46 ± 0.52 | 1.02 ± 0.36 | 0.66 ± 0.31 |
| 2b | 0.84 ± 0.32 | 1.12 ± 0.61 | 1.13 ± 0.41 | 0.90 ± 0.56 |
| 2c | 1.08 ± 0.37 | 1.11 ± 0.60 | 0.83 ± 0.45 | 0.85 ± 0.42 |
| 2ac | 1.50 ± 0.56 | 1.37 ± 0.45 | 1.55 ± 0.72 | 0.80 ± 0.49 |
| 2e | 1.10 ± 0.33 | 1.46 ± 0.70 | 1.25 ± 0.75 | 1.02 ± 0.68 |
| 2f | 1.69 ± 0.46 | 1.30 ± 0.53 | 0.71 ± 0.31 | 0.58 ± 0.22 |
| S | 0.67 ± 0.33 | 1.06 ± 0.37 | 0.48 ± 0.36 | 0.53 ± 0.18 |

**Table S2B**: Distances between tunnel entrances (Å)

| Model | Tunnel | 2b | 2c | 2ac | 2e |
| --- | --- | --- | --- | --- | --- |
| 1R9O1 | 2a | 7.33 ± 0.37 | 22.55 ± 0.71 | 15.74 ± 0.57 | 12.91 ± 0.54 |
| 1R9O2 |  | 9.52 ± 0.64 | 23.07 ± 0.85 | 11.95 ± 0.55 | 13.53 ± 0.72 |
| 1R9O1H |  | 6.99 ± 0.56 | 22.22 ± 0.63 | 15.53 ± 0.54 | 13.05 ± 0.49 |
| 1R9O2H |  | 8.65 ± 0.26 | 20.91 ± 0.51 | 9.76 ± 0.28 | 12.82 ± 0.72 |
|  |  |  |  |  |  |
| 1R9O1 | 2b | x | 20.59 ± 0.51 | 17.11 ± 0.89 | 10.26 ± 0.62 |
| 1R9O2 |  | x | 20.37 ± 0.79 | 14.62 ± 0.83 | 9.73 ± 0.69 |
| 1R9O1H |  | x | 20.61 ± 0.67 | 17.16 ± 0.54 | 10.90 ± 0.79 |
| 1R9O2H |  | x | 19.71 ± 0.39 | 13.47 ± 0.33 | 10.21 ± 0.48 |
|  |  |  |  |  |  |
| 1R9O1 | 2c | x | x | 10.70 ± 0.29 | 10.44 ± 0.35 |
| 1R9O2 |  | x | x | 13.07 ± 0.59 | 10.85 ± 0.53 |
| 1R9O1H |  | x | x | 10.65 ± 0.41 | 9.91 ± 0.32 |
| 1R9O2H |  | x | x | 12.65 ± 0.39 | 9.86 ± 0.44 |
|  |  |  |  |  |  |
| 1R9O1 | 2ac | x | x | x | 9.25 ± 0.52 |
| 1R9O2 |  | x | x | x | 8.76 ± 0.45 |
| 1R9O1H |  | x | x | x | 8.95 ± 0.51 |
| 1R9O2H |  | x | x | x | 8.58 ± 0.36 |
