# Supplementary material for: Structure and Dynamics of the Membrane-Bound Cytochrome P450 2C9
Source: PLoS Comput Biol. 2011 Aug 11;7(8):e1002152. doi: 10.1371/journal.pcbi.1002152 (PMC3154944; doi:10.1371/journal.pcbi.1002152)
Supplement: Protocol S1 — Procedure to construct the models of the soluble CYP2C9. (DOC) [file pcbi.1002152.s014.doc]

**Protocol S1**

**CYP2C9 models**

The flurbiprofen-bound crystal structure of CYP2C9 (pdbid 1R9O, 1.9 Å resolution) contains a soluble form of the enzyme in which the N-terminal transmembrane helix was removed but is otherwise mutation-free. It is closer to the wild-type protein than the warfarin-bound or apo structures (pdbids 1OG5 and 1OG2) {Williams, 2003 #132} which were engineered in the region of the FG loop to increase protein solubility (ref). The 1R9O structure lacks electron density for residues 38-42, preceding helix A, and residues 214-220 in the region of the FG loop. Models 1R9O1 and 1R9O2 were constructed by modeling the missing residues in 1R9O either from the 1OG5 structure (1R9O1) or ab initio (1R9O2). 1R9O2 was built following the observation that the F’ and G’ helices from 1OG5 do not fit in the electron density of the 1R9O structure.

The procedures were as follows:

1R9O1: The mutated residues from 1OG5 were replaced by the wild type sequence. Residues 43-44, 210-213, and 221-228 from 1R9O were removed in order to transfer the structure of the A’, F’ and G’ helices from 1OG5 onto 1R9O.

1R9O2: All residues for which electron density was available in the 1R9O crystal structure were retained. An extended peptide of wild type sequence was inserted between residues 38 and 42. 2000 steps of energy minimization were performed fixing all residues except 37 to 43. Between residues 214 and 220, a peptide of the wild type sequence flanked on each side by two glycines was inserted and optimized by constrained annealing. First, the peptide was annealed for 20 ps. Restraints ensured a good match of the flanking glycines and the corresponding backbone atoms in1R9O (rmsd ~ 0.8 Å). After the removal of the four glycines and the insertion of the peptide in 1R9O, 2000 steps of conjugate gradient minimization followed by 20 ps of annealing were performed. In each case, the annealing protocol was: (i) 1ps of heating to 1000K (thermal bath relaxation time tautp=0.2ps), (ii) 4ps of constant temperature simulation at 1000K (tautp between 1 and 4ps), (iii) 15ps of fast cooling to 0 K (tautp=0.05ps). All residues except 213 to 222 were kept fixed. Finally, the residues 212-214 and 222-224 formed interacting β-strands at the base of the FG loop. The same simulated annealing protocol was subsequently used for converting the linker between the trans-membrane helix and the globular domain from a coarse-grain to an all-atom representation.
