# Supplementary material for: Structure and Dynamics of the Membrane-Bound Cytochrome P450 2C9
Source: PLoS Comput Biol. 2011 Aug 11;7(8):e1002152. doi: 10.1371/journal.pcbi.1002152 (PMC3154944; doi:10.1371/journal.pcbi.1002152)
Supplement: Protocol S3 — Procedure to equilibrate the atomic-resolution models. (DOC) [file pcbi.1002152.s016.doc]

**Protocol S3**

**Atomic resolution simulations: Equilibration procedure**

The equilibration of the soluble models was performed as follows: (i) 11000 steps of conjugate gradient energy minimization with positional restraints on the protein non-hydrogen atoms gradually decreased from 1000 to 0 kcalmol^-1^Å^-2^ , (ii) 25 ps of heating to 300K in the NVT ensemble followed by 200 ps of equilibration in the NPT ensemble with gradually decreasing positional restraints on protein non-hydrogen atoms (force constant 50 ≥ k ≥ 0 kcalmol^-1^Å^-2^), (iii) 1 ns of final equilibration in the NPT ensemble without positional restraints.

The equilibration of the membrane-bound models of CYP2C9 was performed as follows: (i) 11000 steps of energy minimization (same as for the soluble models), (ii) 1.5 ns NPAT (NPT with constant area in the xy plane) simulation at 300K with gradually decreasing positional restraints on protein non-hydrogen atoms (force constant 50 ≥ k ≥ 0 kcalmol^-1^Å^-2^), (ii) 5 ns NPAT simulation, (iii) 2.5 ns NPγT (NPT with imposed surface tension) simulation. The surface tension was either 50 or 60 dyn/cm. Lower values resulted in simulations in which the area per lipid decreased rapidly to unrealistic values (data not shown), as observed previously for the lipid force field parameters used here (Jojart and Martinek, 2007).
